# A systematic review of non-hormonal treatments of vasomotor symptoms in climacteric and cancer patients

**DOI:** 10.1186/s40064-015-0808-y

**Published:** 2015-02-10

**Authors:** Juergen Drewe, Kathleen A Bucher, Catherine Zahner

**Affiliations:** Max Zeller AG, Seeblickstr. 4, 8590 Romanshorn, Switzerland; Bioconsult GmbH, Rickenbach, Switzerland

**Keywords:** Climacteric symptoms, Vasomotor symptoms, Menopause, Non-hormonal treatments, Systematic review

## Abstract

The cardinal climacteric symptoms of hot flushes and night sweats affect 24-93% of all women during the physiological transition from reproductive to post-reproductive life. Though efficacious, hormonal therapy and partial oestrogenic compounds are linked to a significant increase in breast cancer. Non-hormonal treatments are thus greatly appreciated.

This systematic review of published hormonal and non-hormonal treatments for climacteric, and breast and prostate cancer-associated hot flushes, examines clinical efficacy and therapy-related cancer risk modulation.

A PubMed search included literature up to June 19, 2014 without limits for initial dates or language, with the search terms, (hot flush* OR hot flash*) AND (clinical trial* OR clinical stud*) AND (randomi* OR *observational*) NOT *review*). Retrieved references identified further papers. The focus was on hot flushes; other symptoms (night sweats, irritability, etc.) were not specifically screened. Included were some 610 clinical studies where a measured effect of the intervention, intensity and severity were documented, and where patients received treatment of pharmaceutical quality. Only 147 of these references described studies with alternative non-hormonal treatments in post-menopausal women and in breast and prostate cancer survivors; these results are presented in Additional file 1.

The most effective hot flush treatment is oestrogenic hormones, or a combination of oestrogen and progestins, though benefits are partially outweighed by a significantly increased risk for breast cancer development.

This review illustrates that certain non-hormonal treatments, including selective serotonin reuptake inhibitors, gabapentin/pregabalin, and *Cimicifuga racemosa* extracts, show a positive risk-benefit ratio.

**Key points**

- Several non-hormonal alternatives to hormonal therapy have been established and registered for the treatment of vasomotor climacteric symptoms in peri- and post-menopausal women.
- There are indications that non-hormonal treatments are useful alternatives in patients with a history of breast and prostate cancer. However, confirmation by larger clinical trials is required.

**Electronic supplementary material:**

The online version of this article (doi:10.1186/s40064-015-0808-y) contains supplementary material, which is available to authorized users.

## Introduction

Well-characterised primary symptoms associated with the climacterium include hot flushes (HF), sweating, insomnia, nervousness and irritability, palpitations, changes in libido, dyspareunia, depression, musculoskeletal pain, vaginal pruritus and dryness, pain and/or inflammation, and increased bone turnover (Burger et al. 2002; Crandall et al. 2011). These symptoms vary in frequency and severity, and are presumed to be evoked by the physiological decrease in ovarian function as women transition from reproductive to post-reproductive life (Kronenberg 1990; Nakano et al. 2012; Crandall et al. 2011; Hunter et al. 2012). In other patients, the same symptoms may be caused by chemotherapy or surgery relating to breast cancer or other malignancies. The prevalence of the cardinal vasomotor symptoms, HF and night sweating, varies between 24 and 93% in peri- and post-menopausal women (Kronenberg 1990; Nakano et al. 2012; Crandall et al. 2011; Hunter et al. 2012), and is observed in more than 65% of breast cancer patients (Adelson et al. 2005). Patients with chemotherapy-induced ovarian insufficiency experience even more severe symptoms than patients undergoing normal ageing (Adelson et al. 2005; Carpenter et al. 1998), and the effect on the quality of life varies greatly among those affected.

Even men experience HF during normal ageing, evoked by gradually decreasing testosterone levels. One Swedish survey found that 31% of non-castrated ageing men reported HF and, of these, the quality of life was affected in 50% (Spetz et al. 2003). Aggravated symptoms are observed when testosterone concentrations decrease rapidly, such as during anti-androgen treatment or after orchiectomy for prostate cancer (Adelson et al. 2005). About 63%-80% of post-orchiectomy prostate cancer patients complained of long-lasting HF (Charig & Rundle 1989; Karling et al. 1994; Schow et al. 1998). More than 48% of these patients complained of sustained flushes 5 years after onset, and more than 40% experienced flushes even 8 years after castration (Karling et al. 1994).

### Aetiology

Though not fully understood, several authors propose that the aetiology of HF is due to a changed thermoregulation set point of the hypothalamus evoked by the abruptly-lowered oestrogen levels during menopause (Adelson et al. 2005; Kronenberg 1994). Other neuroendocrine hormones (e.g., α-adrenergic mechanisms (Berendsen 2000; Rosenberg & Larsen 1991)) may be involved in this disturbance of temperature regulation. Oestrogen interacts with neurotransmitters, such as norepinephrine and endogenous opioids (Casper & Yen 1985; Rebar & Spitzer 1987), as well as serotonin (Berendsen 2000), and thereby alters the temperature regulation set point in the hypothalamus.

Post-menopausal women show a diminished serotonergic activity compared to pre-menopausal controls. After oestrogen hormone replacement therapy, serotonin activity becomes partly normalised (Blum et al. 1996; Gonzales & Carrillo 1993; Halbreich et al. 1995). Serotonin 5-HT_2A_ receptors play a key role in the development of HF in the hypothalamus. These receptors are up-regulated during oestrogen withdrawal (Biegon 1990). Blockage of 5-HT_2A_ receptors by the 5-HT_2_,_3_ receptor blocker, mirtazapine, in post-menopausal women reduced the frequency and intensity of HF (Waldinger et al. 2000). On the other hand, activation of 5-HT_2_ receptors with *m*-chlorophenylpiperazine, a 5-HT_2A:2C_ receptor agonist, induced post-menopausal symptoms, such as sweating and hot and cold flushes and palpitations (Berendsen 2000) (and references cited therein). The frequency and severity of HF secondary to medical castration for advanced prostate cancer are reduced with sertraline (Roth & Scher 1998). It has also been shown that 5-HT_1A_, 5-HT_1D_ and 5-HT_7_ receptors are also involved in hypothalamic thermoregulation (Hedlund et al. 2003; Naumenko et al. 2011). *Cimicifuga racemosa* extracts (CRE) bind to the serotonin receptors 5-HT_1A_, 5-HT_1D_ and 5-HT_7_ (Burdette et al. 2003; Powell et al. 2008), and a part of its effect on HF may be mediated by these receptors. In addition, oestrogen increases the density of 5-HT_2A_ receptors in the *nucleus accumbens*, suggesting an effect of oestrogen withdrawal on mood and may explain the onset of depressive symptoms in menopause (Fink & Sumner 1996; Fink et al. 1996).

### Treatment of climacteric symptoms with hormone therapy

Different treatments have been established for climacteric symptoms. For many years, hormone therapy (HT) using oestrogen or a combination of oestrogen and progestins was the gold standard. Much evidence exists that HT effectively reduces climacteric symptoms in women (as indicated by a Cochrane meta-analysis of 24 randomised, placebo-controlled trials (MacLennan et al. 2009)).

### HT of climacteric symptoms and risk of breast cancer

As early as 1997, initial epidemiologic evidence showed that HT may increase the risk of breast cancer. One meta-analysis (Collaborative Group on Hormonal Factors in Breast Cancer 1997) of 51 epidemiologic studies comparing data from 52,705 women with breast cancer, and including 108,411 women without breast cancer, revealed that the prevalence of breast cancer was significantly increased in women using HT, and rose with duration of use. Several subsequent studies have confirmed these data.

The large prospective Women’s Health Initiative (WHI) trial that included 16,608 post-menopausal women was prematurely terminated due to a significantly increased risk of breast cancer development (nominal hazard ratios (HR) 1.26 (95% confidence interval (95% CI) 1.00-1.59), coronary heart disease (HR 1.29 (95% CI 1.02-1.63)), stroke (HR 1.41 (95% CI 1.07-1.85)) and venous thromboembolic disease (HR 2.11 (95% CI 1.58-2.82)) in the hormone-treated arm (Rossouw et al. 2002).

Several other prospective und epidemiological studies (Chlebowski et al. 2009; Collaborative Group on Hormonal Factors in Breast Cancer 1997; Beral & Million Women Study Collaborators 2003; Porch et al. 2002; Weiss et al. 2002; Beral et al. 2002) confirmed these findings; for a review, cf. (Collins et al. 2005).

In one large study combining data from a randomised trial (n = 16,608) and an observational study (n = 41,449) in the WHI population, temporal trends of breast cancer diagnosis were analysed in groups who received daily oestrogen plus medroxyprogesterone acetate or placebo (Chlebowski et al. 2003). This study revealed that the breast cancer risk increased steadily throughout the mean follow-up of 5.6 years of the intervention period in both study groups. However, at the end of the intervention phase, hazard-ratio increased significantly for total breast cancer (HR 1.24 (95% CI 1.02-1.50); p < 0.001)) and for invasive breast cancer (HR 1.24 (95% CI 1.01-1.50); p = 0.003) in the oestrogen plus medroxyprogesterone acetate group compared with placebo. Interestingly, the elevated risk in the hormone-treated group decreased rapidly after stopping HT treatment, however, a small and not significantly increased risk (HR 1.15 (95% CI 0.98-1.37)) was still present after 11 years of follow up (Stevenson et al. 2011). The decrease was unrelated to mammography frequency, since this was unchanged in the two groups. These results also concurred with anecdotal evidence that withdrawal of HT alone led to breast cancer regression (Powles & Hickish 1995). A subgroup analysis showed that the breast cancer risk of combined treatment with oestrogen and progestin increased only in the subgroup of patients who received a hormonal treatment prior to the study (adjusted HR 1.96 (95% CI 1.17-3.27), N = 4311) and not among the patients who never used hormonal treatment before (HR 1.02 (95% CI 0.77-1.36); N = 12297) (Anderson et al. 2006).

In the *Million Women Study* (n = 1,084,110), current HT users demonstrated a significantly increased relative risk (RR): 1.66 (95% CI 1.58-1.75) for developing breast cancer, whereas past users had no increased risk (RR 1.01 (95% CI 0.94-1.09)). The breast cancer risk increased with duration of HT treatment and was more pronounced with oestrogen-progestagen combinations and, with respect to receptor status, were mixed and did not show a significant increase in oestrogen receptor-positive cancers (Chlebowski et al. 2003). However, dose or HT preparation (oral vs. transdermal vs. implant) did not affect overall results (Beral & Million Women Study Collaborators 2003). Other studies have shown diverging results: a trend (p = 0.09) towards a lower risk (Prentice et al. 2008) for breast cancer development and significantly lower risk using monotherapy with conjugated equine oestrogen alone compared to combination HT (Ross et al. 2000; Saxena et al. 2010; Beral et al. 2011).

After publication of the WHI studies and the Million Women Study, HT use decreased drastically worldwide (Hersh et al. 2004; Canfell et al. 2008; Antoine et al. 2011). Notably, the lowered use was accompanied by a significant decrease in breast cancer incidence in many countries (Canfell et al. 2008; Ravdin et al. 2007; Canfell et al. 2009) that was more evident in oestrogen-receptor positive than in oestrogen-receptor negative cancers, and in women older than 50 years of age (Ravdin et al. 2007). It was most prominent in countries with a high absolute prevalence of HT use and could not be explained by changes in the mammography rate; cf. review by Zbuk and Anand (Zbuk & Anand 2012). Using epidemiologic data between the years 2000 (118,724 patients) to 2007 (154,447) from Israel, Silverman *et al*. (Silverman et al. 2011) could clearly discriminate between the effect of HT and mammography rate for the risk of breast cancer development and confirmed that the drop in HT frequency caused a parallel drop in the breast cancer rate. After cessation of HT, the increased risk of breast cancer disappeared within 2 years (Narod 2011; Chlebowski et al. 2009; Beral et al. 2011).

### Risk assessment of HT treatment in breast cancer patients

There is a need for medical management of climacteric symptoms in cancer patients. In one survey, only 20.5% of women being treated for breast cancer and suffering from moderate to severe HF actually received any treatment for their symptoms (Gupta et al. 2006). In an earlier study in 190 women with breast cancer, the prevalence of post-menopausal symptoms, such as HF, was 65%; half of the women felt that they needed treatment of these symptoms (Couzi et al. 1995).

The safety of HT in breast cancer patients was reviewed in 20 studies by Antoine *et al*. (Antoine et al. 2007). Citing 10 prospective and two randomised studies that were heterogeneous with respect to tumour characteristics, prognostic factors and therapies, the authors conclude that there are no reassuring data indicating that HT is not without risks. Antoine also cited two studies that showed decreased recurrence rates and two others with lowered breast cancer mortality under HT treatment (Antoine et al. 2007). Nevertheless, one randomised study (HABITS trial, n = 434) showed that HT increased the recurrence of breast cancer significantly (relative HR 3.3; 95% CI 1.5-7.4; p = 0.02) (Holmberg & Anderson 2004), and was therefore prematurely terminated. The other randomised study (von Schoultz & Rutqvist 2005) (Stockholm trial, n = 378) did not show any difference in the cancer recurrence rate between HT and the control group (relative HR 0.82; 95% CI 0.35-1.9). Since the overall analysis of both studies revealed a significantly greater risk of cancer recurrence in the HT group (relative HR 1.8; 95% CI 1.03-3.1), the Stockholm trial was likewise discontinued. The authors therefore concluded that guidelines should advise against using HT in patients with a history of breast cancer (Antoine et al. 2007).

Progestogens are effective in treating post-menopausal symptoms: In one open (Erlik et al. 1981) and one prospective randomised trial (Loprinzi et al. 1994a), more than 80% of patients showed symptom improvement after receiving megestrol acetate. The latter study also included 66 men with prostate cancer who complained of HF after androgen deprivation therapy. The degree of symptom relief was similar in women and men. However, the safety of progestagens has not been established.

## Treatment with synthetic compounds with partial estrogenic activity

The minimum requirements for the clinical evaluation of new products in the treatment of vasomotor symptoms have been defined by the Food and Drug Administration (FDA) (FDA. U.S. Department of Health and Human Services Food and Drug Administration 2003) and the European Medicines Agency (EMA) (EMEA. Committee for Medicinal products for human use (CHMP) 2005). Besides the definition of outcome parameters and the methods of their evaluation, these guidelines set standards requiring at least 12 weeks of treatment for a randomised controlled clinical trial. This considers the need for chronic treatment in this indication. Many of the academia-driven studies have been performed with shorter treatment durations, and could therefore be considered as providing merely supportive evidence.

### Tibolone

Tibolone is a synthetic steroid gonadomimetic with weak oestrogenic, androgenic and progestogenic properties (Baber et al. 2005). Its action has been described as a **s**elective **t**issue o**e**strogenic **a**ctivity **r**egulator (STEAR) (Kloosterboer 2004), meaning that the amount of tissue enzymes (sulfatase, sulfotransferase, and 17β-hydroxysteroid dehydrogenase) control the sum of active ligands for steroid receptors, and may be responsible for tissue-specific effects. This concept may also explain certain discrepancies from *in vitro* investigations: *In vitro,* tibolone exerts a proliferative effect on an oestrogen–receptor-positive breast cancer cell line (MCF-7), indicating a potential tumour promoting effect (Lippert et al. 2002; Mueck et al. 2003). *In vivo*, the proliferative effects of tibolone were investigated in a randomised controlled trial by measuring the content of the nuclear antigen Ki-67 (a proliferation marker) in breast tissue biopsies after treatment with tibolone, oestradiol/norethisterone acetate or placebo. No increase in proliferation was seen in the tibolone or placebo group, whereas a significant increase was observed in the oestradiol/norethisterone acetate group (Conner et al. 2004). This was further investigated in a randomised, placebo-controlled study in patients with oestrogen receptor-positive primary breast cancer (Kubista et al. 2007). However, the treatment duration was only 14 days and only a trend of a beneficial effect on proliferation was observed.

Several studies have investigated tibolone as a potential alternative to classical HT to reduce climacteric symptoms (Landgren et al. 2002; Hammar et al. 1998). One international study conducted at 38 sites throughout Europe, South Africa and Mexico with 485 women compared tibolone (2.5 mg/d) to desvenlafaxine (100 mg/d) and placebo, examining reduction in the number of HF. No difference was noted between desvenlafaxine and placebo, but tibolone significantly reduced the number of HF when compared to placebo (p < 0.001), though there was a significant increase in the number of tibolone subjects who experienced bleeding (p < 0.024) (Bouchard et al. 2012).

In a large randomised study in 4,538 post-menopausal women (Cummings et al. 2008), the effect of tibolone was compared to placebo over 34 months with regard to bone fracture, breast cancer development and cardiovascular diseases. Compared to placebo, patients in the tibolone group had a decreased risk of vertebral fractures (p < 0.001), a decreased risk of non-vertebral fractures (p < 0.01), and a decreased risk of developing invasive breast (p = 0.02) and colon cancer (p = 0.04). On the other hand, patients had an increased risk of stroke (p = 0.02), for which the study was prematurely terminated.

Tibolone’s symptom-alleviating effects have also been demonstrated in breast cancer survivors in several clinical studies (Baber et al. 2005; Kroiss et al. 2005). In one prospective study, 103 post-menopausal women received either tibolone, oestrogen plus medroxyprogesterone acetate (i.e., HT) or placebo for one year (Marchesoni et al. 2006). An increase in mammographic breast density (as a surrogate marker for the development of breast cancer) was observed in 45% after HT, but only in 2.3% after tibolone treatment, and none in the placebo group (Marchesoni et al. 2006; Greendale et al. 1999). However, a later study did not confirm these results (Kutlu et al. 2004).

The safety and efficacy of tibolone have been studied in great depth in the large LIBERATE (Livial Intervention following Breast Cancer; Efficacy, Recurrence And Tolerability) trial (Kenemans et al. 2009) in 3,148 women with breast cancer and severe climacteric symptoms due to treatment with tamoxifen, aromatase inhibitors, gonadotropin-releasing hormone analogues or chemotherapy. Patients were randomised to receive either 2.5 mg/d tibolone or placebo. The primary endpoint was the rate of breast cancer recurrence. After a median follow-up of 3.1 years, patients in the tibolone group had a significantly higher recurrence rate (p = 0.001, HR 1.40 (95% CI 1.14-1.70)) than after placebo treatment. Vasomotor symptoms and bone-mineral density improved significantly with tibolone compared to placebo. From the finding that breast cancer recurrence was more evident in patients with oestrogen-receptor positive tumours, the authors concluded that tibolone exerted an oestrogenic effect, and that its use in patients “with a known, past or suspected breast cancer will remain contraindicated”. The LIBERATE trial bone sub-study recruiting 763 women confirmed these results with the clarification that the increase in breast cancer recurrence and tibolone use was higher in women having normal bone mineral density than in those with lower bone density (Bundred et al. 2012).

## Non-hormonal alternatives to HT in the treatment of climacteric symptoms

Some of the non-hormonal treatments (e.g., isoflavones and vitamins) are marketed as food supplements in different countries (Europe, USA and Canada). Cimicifuga racemosa is currently registered as a herbal medicinal drug for the treatment of climacteric complaints in several European countries (among them, Austria, Belgium, Czech Republic, Denmark, Finland, Germany, Hungary, Sweden, Switzerland, and UK) and Australia, South Korea, South Africa. In some countries (such as USA) Cimicifuga racemosa is used as a food supplement. Paroxetine is registered in the USA for treatment of climacteric complaints. Registration of desvenlafaxine was rejected in the USA, and a New Drug Application (NDA) was withdrawn by the manufacturer in Europe. So far as we know, none of the other mentioned compounds are registered for the treatment of menopausal symptoms.

### Literature search methodology

The search was carried out using the PubMed database and included all literature up to June 19, 2014. No limits were set for language. References cited in the papers retrieved were used to locate further papers. The following search terms were used: (hot flush* OR hot flash*) AND (clinical trial* OR clinical stud*) AND (randomi* OR observational) NOT review). This search revealed 609 clinical studies. Only 147 of these references described studies with alternative, non-hormonal treatments for HF in post-menopausal women and in breast cancer survivors, and these are presented in Additional file 1. All effort was made to include statistical relevance (p values, confidence intervals) whenever possible. It was noted that, in many of the earlier studies (prior to 2000), this informative criterion was less rigorously provided.

### Dopamine agonists

As mentioned above (Berendsen 2000; Rosenberg & Larsen 1991), it is thought that α-adrenergic mechanisms participate in the pathophysiology of HF. And as is often the case in medicine, anecdotal reports pique interest in the testing of a specific drug for a new indication. In this instance, hypertensive women being treated with methyldopa, an α-adrenergic, centrally-active sympatholytic, reported improvement in HF symptoms. In two randomised, placebo-controlled trials each lasting 4 weeks and involving 40 post-menopausal women each, respectively (Nesheim & Saetre 1981; Andersen et al. 1986), significant improvement in HF symptoms was reported. Another study expounded on this and compared the following: bromocriptine (dopaminergic), liposom (indirect dopaminergic), veralipride (antidopaminergic), domperidone (a peripheral antidopaminergic) and placebo for treating vasomotor symptoms of menopause (Zichella et al. 1986). As all active treatments proved effective, different pathways were postulated: A direct action would be expected from dopamine, and direct and indirect dopaminergic agents, while the antidopaminergic drugs are thought to evoke a secondary dopamine-like activity via the short-loop feedback exerted by hyperprolactinaemia on tuberoinfundibular dopamine neurons, or perhaps stimulation of the opioid system (Zichella et al. 1986). Though improvement in HF was seen, concerns about side effects (depression, anxiety, decreased alertness, lethargy, dizziness, headaches, myalgia, etc.) led to other avenues being pursued.

### Adrenergic agonists

Clonidine, as a centrally-active antihypertensive and α_2_-adrenoceptor agonist, seemed promising for the treatment of HF.

Effects of oral clonidine were tested against no treatment in 30 women where significant improvement in HF frequency, severity and duration were noted (Chow et al. 1993).

In a small randomised prospective double-blind study (n = 29), *transdermal* therapy with clonidine (corresponding to 0.1 mg/d) over 8 weeks significantly reduced the number (80%, p < 0.04), severity (73%, p < 0.04) and duration (67%, p < 0.03) of HF, compared to 36%, 29% and 21% for placebo, respectively (Nagamani et al. 1987).

In two larger randomised, double-blind, placebo-controlled cross-over trials in post-menopausal patients, significant improvements in the number, severity and duration of HF were observed: In the first study (n = 100), patients received *oral* clonidine in doses ranging from 0.025 to 0.075 mg b.i.d. for 4 weeks; effects were then compared to placebo (Clayden et al. 1974). In the second study (n = 66), patients received a fixed oral dose of 0.050 mg clonidine or placebo twice daily for 4 weeks (Edington et al. 1980), here however, more adverse events (AEs) were observed in the clonidine vs. placebo groups (dry mouth: 11 vs. 4, insomnia: 8 vs. 4). Since the reduction in HF frequency was small although statistically significant, the authors concluded that clonidine was a medication “that makes flushing more tolerable”.

The effect of low-dose oral clonidine therapy (up to 0.4 mg/day) for up to 4 weeks was further investigated in several other small studies (n = 10-30); results showed either a significant reduction in the number and severity of HF (Laufer et al. 1982; Chow et al. 1993) or no effect (Wren & Brown 1986), but again, in very small patient numbers and thus of limited value.

Positive effects were confirmed in a larger randomised double-blind cross-over study in 110 female *breast cancer survivors* receiving concomitant tamoxifen treatment (Goldberg et al. 1994), where transdermal clonidine (equivalent to a daily oral dose of 0.1 mg) or placebo was given for 4 weeks. Clonidine therapy reduced HF frequency (20% lower than baseline values, p < 0.0001) and severity (10% lower than baseline values, p = 0.02), but accompanied by more AEs (dry mouth, p < 0.001; constipation, p < 0.02; itchiness under the patch, p < 0.01 and drowsiness, p < 0.05). Transdermal clonidine (corresponding to 0.1 mg/d) was also studied in 70 male *prostate cancer survivors* where no significant decrease in HF was noted (p = 0.57) (Loprinzi et al. 1994b).

In a larger trial, the effects of oral clonidine were assessed in 194 post-menopausal women with breast cancer who were receiving tamoxifen treatment (Pandya et al. 2000). After 8 weeks of treatment, frequency of HF decreased by 38% in the clonidine group compared to 24% in the placebo group (p = 0.006); however, patients receiving clonidine reported more sleep disturbances (41% vs. 21%; p = 0.02).

In another double-blind, randomised phase III study that compared clonidine and venlafaxine in 80 breast cancer survivors, most still receiving endocrine treatment, the primary end-point was defined as the frequency of hot flushes after 4 weeks of treatment using HF and other symptom questionnaires. HF frequency was −7.6 HF for venlafaxine and −4.85 for clonidine (p = 0.025). Four clonidine and six venlafaxine patients discontinued due to side effects (Loibl et al. 2007). And in another venlafaxine vs. clondine double-blind, cross-over study in 60 breast cancer survivors, clonidine showed fewer side effects and a higher reduction in HF (55%) when compared with venlafaxine (49%), though statistical significance was not reached. Discontinuation due to side effects was observed in 5/53 for clonidine, and in 14/59 for venlafaxine (p = 0.038) (Buijs et al. 2009).

In a recent double-blind, placebo-controlled trial, 102 patients with a history of breast cancer were randomly assigned (2:2:1) to oral daily doses of either venlafaxine 75 mg, clonidine 0.1 mg, or placebo for 12 weeks (Boekhout et al. 2011). Results from the average daily HF scores were assessed both at the week 12 time point, and over the entire 12 weeks. During week 12, hot flush scores were significantly lower in the clonidine group versus placebo (p = 0.03), but not significant between venlafaxine and placebo (p = 0.07), though the median HF scores were identical in the clonidine and venlafaxine groups at this point. However, over the course of the 12-week trial, there were significant differences between both treatments and placebo (p < 0.001 for venlafaxine vs. placebo; p = 0.045 for clonidine vs. placebo). Frequencies of nausea (p = 0.02), constipation (p = 0.04), and severe appetite loss were higher in the venlafaxine group.

Depending on the study, clonidine is either superior or inferior to venlafaxine as an effective treatment in the management of HF in patients with breast cancer, though not without AEs, such as insomnia, constipation or dry mouth. Other studies are discussed under venlafaxine.

No information is yet available as to whether clonidine treatment can modulate the risk of breast cancer recurrence. On one hand, *in vitro* studies from a group in Argentina have demonstrated a proliferative effect (increase in thymidine incorporation) of clonidine in MCF-7 breast cancer cells (Vázquez et al. 1999), in the mouse mammary tumour cell line MC4-L5 (Bruzzone et al. 2008) and in stromal fibroblasts (Bruzzone et al. 2011). In a case-controlled study using data from 2,079 patients who received clonidine treatment (Friedman et al. 2011), a slight but not significant increase in breast cancer development was observed (odds ratio (OR) 1.08 (95% CI 0.98–1.20)). Based on the widespread use of clonidine, the lack of significant epidemiological data suggests that these results are most likely without clinical relevance.

### Gabapentin/pregabalin

The first evidence that gabapentin/pregabalin exerted a beneficial effect on HF was reported in 2000 by Guttuso (Guttuso 2000), based on results from six patients. Hypothesised mechanisms of action in HF amelioration are modulation of calcium currents and mitigation of hypothalamic tachykinin activity (Baber et al. 2005).

In a further uncontrolled pilot study (Loprinzi et al. 2002a) in 24 post-menopausal women of whom 20 were evaluable, gabapentin was given in doses of 300 to 900 mg/day. With four drop-outs due to AEs (e.g. light-headedness and dizziness), the 16 remaining subjects reported a mean reduction in HF frequency and score of 66% and 70%, respectively.

In a randomised, double-blind, placebo-controlled trial in 59 post-menopausal women with seven or more HF per day, the effects of 900 mg oral gabapentin on HF frequency were assessed after 12 weeks of treatment. Gabapentin evoked a 45% reduction in HF frequency and a 54% reduction in the HF composite score compared to the placebo response (29% (p = 0.02) and 31% (p = 0.01), respectively). In an extension phase, patients were studied in an *open-label trial* where the dose of gabapentin could be increased up to 2700 mg/day, as needed. Treatment with the higher dose showed a further reduction of 54% and 67%, respectively. Common AEs in the gabapentin group were somnolence (n = 6), dizziness (n = 4) and rash with and without peripheral oedema (n = 2), which were not observed in the placebo group. Four patients in the gabapentin group withdrew their consent and terminated participation because of dizziness, rash, heart palpitations and peripheral oedema, respectively. Two patients temporarily reduced the gabapentin dose due to dizziness and sleepiness. In the extension phase, two patients previously in the placebo arm withdrew from the study due to dizziness and peripheral oedema (Guttuso et al. 2003).

In another randomised, double-blind, placebo-controlled, parallel group trial including 60 women with post-menopausal symptoms, the effect of gabapentin (titrated to 2400 mg/day) was compared to conjugated oestrogens (0.625 mg/day) and placebo for the treatment of moderate-to-severe HF (Reddy et al. 2006) with 20 women per arm. Both active treatments showed a significant and comparable reduction in mean HF composite score vs. placebo after 12 weeks of treatment: oestrogen (72%, p = 0.016) and gabapentin (71%, p = 0.004). In the placebo group, the mean HF composite score decreased by 54%. In the gabapentin group, slightly more AEs of headache, dizziness and disorientation were observed.

In one randomised trial (Loprinzi et al. 2007) including 118 patients having HF symptoms that were insufficiently-controlled with antidepressant therapy alone (primarily venlafaxine or paroxetine), adding up to 900 mg/d gabapentin resulted in a 54% (95% CI, 34% to 70%) and 56% (95% CI, 26% to 71%) median reduction in HF frequency and score, which was not statistically better than gabapentin treatment alone, 49% (95% CI 26% to 58%) and 60% (95% CI, 33% to 73%), respectively.

In a randomised, double-blind, placebo-controlled trial in 200 menopausal women, the effect of 3 × 300 mg gabapentin on vasomotoric symptoms was studied over 4 weeks (Butt et al. 2008). Significant decreases for gabapentin over placebo were noted in both the HF score (51.0% vs. 26.5%, p < 0.001) and frequency (45.7% vs. 24.7%, p < 0.001) for the gabapentin vs. placebo groups, respectively. However, gabapentin treatment was accompanied by a significantly higher rate of AEs than placebo in the first treatment week, but these later abated (dizziness: 18% vs. 1%; unsteadiness: 14% vs. 1%, and drowsiness: 12% vs. 1%).

In two recent randomised placebo-controlled trial in 60 and 50 menopausal women, the effect of gabapentin on vasomotoric symptoms were further confirmed: In the first study (Saadati et al. 2013), when given over 12 weeks, 900 mg gabapentin significantly decreased both HF frequency and severity (both p < 0.001). In the second study (Agarwal et al. 2014), the results were confirmed in 50 post-menopausal women after 12 weeks treatment, which was then extended to 24 weeks. Impressive reductions in HF frequency (59.1% and 60.6%) compared to placebo were noted at 12 (p = 0.008) and 24 weeks (p = 0.005), and the composite score decreased by about 80% already at 12 weeks, which continued until the end of the study (both p = 0.001).

A different, gastrorententive galenic formulation of gabapentin was studied in a large randomised, placebo-controlled study in 600 menopausal patients (Pinkerton et al. 2014), providing a continuous drug release in the upper small intestine for 8 to 9 hours. Gabapentin was given asymmetrically (600 mg in the morning and 1200 mg in the evening) over 12 weeks, after which the HF frequency (p = 0.0007) and severity (p = 0.012) decreased modestly, but significantly compared to placebo. These effects were maintained up to 24 weeks (p = 0.0174 and p = 0.0457, respectively). Slightly more (5%) women under gabapentin than placebo withdrew because of AEs (16.7% and 11.5%, respectively). Most common in the start phase were dizziness (12.7% and 3.4%), headache (9.3% and 8.1%) and somnolence (6.0% and 2.7%), which levelled off to comparable values over the study period.

Another double-blind RCT (Loprinzi et al. 2010) was performed using pregabalin to treat HF in 207 post-menopausal women. Two doses (2×75 mg/d and 2×150 mg/d) of pregabalin were compared to placebo for a treatment period of 6 weeks. The HF score decreased significantly by 50%, 65% (p = 0.009) and 71% (p = 0.007) for the placebo, 75 mg b.i.d. and 150 mg b.i.d. group, respectively.

#### Breast cancer

In a pilot study involving 22 breast cancer survivors receiving tamoxifen therapy, HF were treated with 3×300 mg daily gabapentin for 4 weeks. HF duration decreased by 73.6% (p = 0.027) frequency by 44.2% (p < 0.001), and severity by 52.6% (p < 0.001). Four women dropped out due to AEs (nausea, rash, somnolence), while 8/16 women who finished the study showed a complete response (Pandya et al. 2004).

A large study (Pandya et al. 2005) in 420 breast cancer survivors (300 mg/d or 900 mg/d GP vs. placebo over 8 weeks) also confirmed the effect in this patient population: After 8 weeks of treatment, only the 900 mg dose showed a significant reduction in HF frequency (44%, p < 0.0001) and severity (46%; p < 0.0001) versus 15% and 15% in the placebo group. However, the study duration was too short to assess whether the treatment modulated the risk of tumour recurrence.

In a smaller cross-over RCT (N = 66) Bordeleau *et al*. (Bordeleau et al. 2010) showed that gabapentin (up to 900 mg/d) and venlafaxine (up to 75 mg/d) demonstrated a similar 66% reduction in HF score in breast cancer survivors (p < 0.001); 32% of patients preferred gabapentin, while 68% venlafaxine. The latter showed more nausea, appetite loss, constipation, and reduced negative mood changes than gabapentin, whereas gabapentin demonstrated more dizziness and increased appetite compared with venlafaxine (all p < 0.05).

#### Prostate cancer

Two studies investigated the effect of gabapentin in prostate cancer survivors undergoing androgen deprivation therapy: In a double-blind, placebo controlled trial (Loprinzi et al. 2009) in 223 men, slight to moderate and dose-dependent effects on HF frequency and severity could be demonstrated in a short-term study over 4 weeks. Compared to baseline, HF frequency and score decreased by 21.5 (95% CI: 11.3-30.9%) and 27.0% (95% CI: 12.1-36.1%) after placebo and by 45.5% (95% CI: 31.1-50.6%) and 44.4% (95% CI: 35.2-56.3%) after administration of 900 mg/d, respectively. Only in the highest dose could a significantly greater reduction in HF frequency (p = 0.02) compared to placebo be demonstrated. In an extension of the above-cited study (Moraska et al. 2010), 147 patients were either switched from placebo to gabapentin or continued gabapentin treatment for eight additional weeks with doses of gabapentin titrated up to 900 mg/d. The treatment was well tolerated. Effects of previous high dose treatments were maintained and those of low dose and placebo treatments were improved. The majority of the patients opted to take a dose of 600 mg/d. However, no data were obtained regarding the question of whether gabapentin was able to modulate prostate cancer disease.

In summary, results from several randomised controlled trials (RCTs) in post-menopausal women without cancer (Loprinzi et al. 2002a; Guttuso et al. 2003; Reddy et al. 2006; Loprinzi et al. 2007; Butt et al. 2008; Saadati et al. 2013; Agarwal et al. 2014; Pinkerton et al. 2014; Loprinzi et al. 2010) and in breast and prostate cancer survivors (Pandya et al. 2004; Pandya et al. 2005; Bordeleau et al. 2010; Loprinzi et al. 2009; Moraska et al. 2010), gabapentin showed no or a slight effect at lower doses (<900 mg/d GP) or moderate effects at higher doses, ranging from 44% (Pandya et al. 2005) to 71% (Loprinzi et al. 2010; Reddy et al. 2006). The study durations were, in general, short (4–8 weeks) except in three studies (Guttuso et al. 2003; Reddy et al. 2006; Saadati et al. 2013) that continued for 12 weeks and two studies for 24 weeks (Agarwal et al. 2014; Pinkerton et al. 2014).

## Antidepressant drugs

Various antidepressant drugs (e.g., selective serotonin reuptake inhibitors (SSRIs) and serotonin-norepinephrine reuptake inhibitors (SNRIs)) have been studied to treat post-menopausal symptoms. The rationale of using antidepressant drugs is two-fold: Firstly, many patients with climacteric symptoms suffer from depressive symptoms and secondly, antidepressant drugs acting on synaptic serotonin concentrations may beneficially interfere with the pathophysiology of HF (Burdette et al. 2003; Hedlund et al. 2003; Naumenko et al. 2011).

### Selective serotonin reuptake inhibitors (SSRI)

#### Paroxetine

In 2003, Stearns and colleagues performed a large randomised, double-blind, placebo-controlled, parallel group study in 165 post-menopausal women administering placebo or 12.5 mg/d or controlled-release 25.0 mg/d for 6 weeks (Stearns et al. 2003). The mean HF frequency (and median composite score) decreased by 37.8% (1.8), 62.2% (3.3) and 64.6% (3.2) in the placebo, 12.5 mg/d and 25 mg/d paroxetine groups, respectively. Although not statistically significant, AEs (mainly headache, dizziness, nausea, and insomnia) were predominantly observed with the higher paroxetine dose. This same group confirmed these results in 2005 in a double-blind, cross-over, placebo-controlled study involving 151 post-menopausal women. Women received first paroxetine (10 or 20 mg/d) or placebo for 4 weeks, and then the other treatment option (Stearns et al. 2005). The 10 mg dose reduced the hot flush frequency and composite score by 40.6% and 45.6% vs. 13.7% and 13.7% for placebo, respectively (p = 0.0006 and p = 0.0008) and 20 mg paroxetine by 51.7% and 56.1% vs. 26.6% and 28.8% for placebo (p = 0.002 and p = 0.004, respectively). While efficacy was similar with the two paroxetine doses, women were less likely to discontinue the low dose paroxetine, which was also associated with a significant improvement in sleep compared to placebo (p = 0.01).

Simon *et al*. reported the results of two large RCTs in a total of 1184 menopausal women (Simon et al. 2013). Both studies evaluated the effect of 7.5 mg daily paroxetine or placebo, one study (N = 614) over 12, the other (N = 570) over 24 weeks. In the 12-week study, HF frequency decreased significantly more from baseline (−33% vs. -23.5%; p < 0.0001) at week 4, and week 12 (−43.5% vs. -37.3%; p = 0.009). However, HF severity score decreased significantly only at week 4 (-0.09 vs. -0.05; p = 0.0048) but not at week 12 (−0.10 vs. -0.09; p = 0.2893). In the 24-week study, HF frequency decreased significantly more from baseline (−28.9% vs. -19.0%; p < 0.0001) at week 4, and at week 12 (−37.2% vs. -27.6%; p = 0.0001. The severity score decreased significantly at week 4 (−0.09 vs. -0.06; p = 0.0452) and week 12 (−0.12 vs. -0.07; p = 0.0114). Final results for HF frequency and severity at week 24 were not provided.

In 2013, Huang *et al.* (Huang et al. 2013) compared paroxetine alone to paroxetine plus isopropanolic *Cimicifuga racemosa* extract. Results from the Kupperman (HF frequency and severity) and Hamilton depression scales (HAMD) for the combined treatment were superior with the combined treatment vs. paroxetine alone (p < 0.01 and p < 0.05, respectively).

##### Breast cancer

In 2000, Stearns and colleagues investigated the effect of paroxetine in an observational pilot study in 30 women with prior breast cancer over a treatment period of 5 weeks at a dose of 10 mg/day for one week, followed by 20 mg daily (Stearns et al. 2000). The HF frequency was reduced by 67% and severity by 75% (95% CI: 56%-79% and 66%-85%, respectively). Though somnolence was observed in four patients leading to drug discontinuation in two patients and likewise dose reduction in two, there was a statistically significant improvement in depression, sleep disturbances, anxiety, and quality of life scores.

#### Sertraline

In a double-blind, placebo-controlled, crossover trail in 102 menopausal women, sertraline 50 mg/d or placebo was given over 4 weeks (Gordon et al. 2006). The number of hot flushes was significantly lower during sertraline treatment than placebo (p = 0.002): mean reduction for the difference of 2.8 (95% CI 0.8-4.9; p = 0.007 during daytime and 2.3 (95% CI 0.3-4.2; p = 0.03) during nighttime. The severity of hot flushes was not significantly different between the treatments, but there was a significant improvement in HF score (p = 0.001) on sertraline. However, although significant, the overall benefit was comparatively small.

No significant effect in alleviating HF frequency and intensity better than placebo was found in a blinded, placebo-controlled RCT involving 99 menopausal women taking 50 or 100 mg sertraline daily for 6 weeks (Grady et al. 2007). Inconclusive results were observed in a further placebo-controlled RCT in 102 women where HF symptoms and severity score improved over nine weeks in one-third of the women, one-third showed no change, and one-third worsened (Kerwin et al. 2007). In another placebo-controlled RCT in 44 menopausal women (Aedo et al. 2011), a higher percentage of patients (p = 0.01) showed a symptomatic improvement of climacteric symptoms (e.g., 35.3% placebo and 81.3% sertraline).

##### Breast cancer

Two studies investigated sertraline in women at high risk of, or having, breast cancer and on adjuvant tamoxifen therapy. Kimmick studied 62 women who received either 50 mg sertraline/day or placebo for 6 weeks in a randomised, double-blind, cross-over design. Treatment effects were only small: a 50% decrease in HF frequency was observed in 36% of the sertraline patients versus 27% receiving placebo (p = 0.7); (Kimmick et al. 2006). Wu (Wu et al. 2009) compared 25–100 mg/d vs. placebo in 65 women who had either had, or were at high risk for, breast cancer in a 6-week study (4-week treatment phase). No significant differences were noted in either the HF frequency or severity score between the groups.

In five case reports, Roth *et al*. (Roth & Scher 1998) showed sertraline improved HF symptoms in **prostate cancer survivors** who were also treated for concomitant depression.

#### Fluoxetine

In a placebo-controlled, double-blind study with a follow-up period of 36 weeks, 150 healthy women with menopausal symptoms were randomised into three groups receiving placebo, fluoxetine, or citalopram (Suvanto-Luukkonen et al. 2005). The initial 10 mg SSRI doses (for fluoxetine and citalopram) increased to 20 mg after 4 weeks and to 30 mg after 24 weeks. The main outcome measures were HF frequency and Kupperman index. No significant differences were observed in the number of HF or severity index between placebo and both SSRIs. The authors concluded that citalopram and fluoxetine cannot be recommended for treating menopausal complaints when vasomotor symptoms are the primary problem.

In 2007, Oktem and colleagues compared 20 mg fluoxetine with 40 mg *Cimcifuga racemosa* extract (CRE) in 120 menopausal women. After 24 weeks, the HF symptom score were significantly reduced in the CRE group (85% vs. 62%), and night sweats were likewise fewer with CRE (both p < 0.01).

##### Breast cancer

A modest treatment effect was obtained in 81 female **breast cancer survivors**. Patients were treated with fluoxetine, 20 mg daily, or placebo over 4 weeks in a randomised, double-blind. cross-over design (Loprinzi et al. 2002b) and demonstrated a 50% decrease in HF score in the fluoxetine versus 36% in the placebo arm (p = 0.02).

#### Citalopram

In a placebo-controlled, double-blind study (cf, Section Fluoxetine, (Suvanto-Luukkonen et al. 2005)), no significant differences were observed in the number of HF or severity index among citalopram, fluoxetine and placebo.

More positive, dose-independent results were obtained in another placebo-controlled trial involving 254 post-menopausal women and 10, 20, or 30 mg/d citalopram (the same dose range as in the study above (Suvanto-Luukkonen et al. 2005)) given for 6 weeks and compared to placebo (Barton et al. 2010). From baseline, reductions in HF frequency (46%, 43% and 50%, p ≤ 0.001) and mean HF scores (49%, 50%, 55%, p ≤ 0.002) for the respective increasing citalopram were superior to placebo (20% and 23%, respectively). Citalopram was well-tolerated without any significant AEs.

##### Breast cancer

In a four-week study, citalopram was investigated in 26 breast cancer survivors at doses of 10 mg during week 1, and 20 mg for weeks 2–4. A 58% reduction in HF frequency and a 64% reduction in HF score from baseline were reported (Barton et al. 2003).

#### Escitalopram

In a small, uncontrolled pilot study in 25 menopausal women, the effect of escitalopram was assessed at doses of 10–20 mg/d (flexibly dosed for 8 weeks) in treating HF. Women reported a significant decrease in HF frequency (52.2%) and severity (53.8%) from baseline (both p = 0.0001). With a responder being defined by at least a 50% decrease in HF frequency (Defronzo Dobkin et al. 2009), 16 patients were treatment responders, with an average decrease of 55%.

These results were partly contradicted in two small (N = 16 and N = 26) double blind, placebo-controlled pilot studies (Freedman et al. 2011). Escitalopram was given at a dose of 10 mg/d (study 1) or 20 mg/d (study 2) over 8 weeks. Escitalopram at 10 mg or 20 mg/day was not effective in treating menopausal HF.

Expanded results from one randomised, double-blind, placebo-controlled, parallel arm trial for 8 weeks in 205 women with menopausal symptoms reported the efficacy and tolerability of 10–20 mg/day escitalopram on HF frequency, severity and bother (Freeman et al. 2011; Carpenter et al. 2012; Ensrud et al. 2012). At baseline, HF frequency was 9.78/day (SD 5.60) and, at week 8, was significantly less in the escitalopram group versus placebo (−4.60 vs. –3.20; p = 0.004). In the escitalopram group, 55% (versus 36% in the placebo group) reported ≥50% decreases in HF frequency (p = 0.009), and significant decreases in HF severity were likewise noted (p = 0.003). New complaints reported by >10% in the escitalopram group included dizziness/light-headedness (14%), vivid dreams (13%), nausea (11%) and hyperhydrosis (11%). Some 4% (7 escitalopram, 2 placebo) discontinued the study due to side effects.

#### Venlafaxine

After the exploitation of the SSRIs (e.g., paroxetine, sertraline, fluoxetine, citalopram and escitalopram) in treating vasomotor symptoms, the SNRIs (e.g., venlafaxine, desvenlafaxine) were put to the test. One study reported on 80 post-menopausal patients who were randomised to receive either placebo or 37.5 mg extended-release daily venlafaxine for one week, followed by 11 weeks of 75 mg venlafaxine. Although there was a trend toward lower HF severity scores in the treatment group, the difference between treatments did not reach significance (p = 0.25). Three AEs, dry mouth, sleeplessness, and decreased appetite, were significantly more frequent in the venlafaxine group (Evans et al. 2005).

Loprinzi *et al.* compared two doses of oral venlafaxine (37.5 mg/d and 75 mg/d) to i.m. medroxyprogesterone acetate (MPA) in 218 post-menopausal women. MPA was superior in all aspects, including lower toxicity: A 50% decrease in HF frequency was achieved by 46% in the VEN group, and 74% in the MPA group; furthermore, a 55% decrease in the HF score was noted for VEN, while this result was 79% for MPA (all p < 0.0001) (Loprinzi et al. 2006).

##### Breast and prostate cancer

In spite of genetic variations among patients, it has been shown that venlafaxine is a weaker inhibitor of cytochrome P450 2D6 (CYP2D6) than paroxetine, and thus only slightly reduces plasma concentrations of endoxifen, the potent tamoxifen metabolite (Pritchard 2010) (This is discussed in further detail under 4.2, Safety of Antidepressants in Patients with a History of Breast Cancer, and 4.2.3, Interactions with Tamoxifen). Extrapolating, venlafaxine could thus potentially be preferable to SSRIs in the treatment of cancer survivors suffering from HF.

Loprinzi tested this hypothesis using velafaxine in breast (82%) and prostate cancer survivors (18%). After 4 weeks of treatment, 12.5 mg b.i.d. venlafaxine was shown to be effective in reducing - by at least half - the frequency of vasomotor symptoms in 54% of patients; and 58% reported a median 55% reduction in the HF score (95% CI 22-71%). Three patients terminated participation – two due to AEs (decreased concentration, depression, nausea, dry mouth, fatigue, and sleepiness) and 1 switched treatment to megestrol acetate (Loprinzi et al. 1998).

Carpenter tested venlafaxine in breast cancer survivors in two sequential, double-blind, placebo-controlled cross-over trials using low, 37.5 mg/d (n = 57) and high doses, 75 mg/d (n = 20) (Carpenter et al. 2007). Compared to placebo, both doses significantly reduced HF frequency (42% and 25% (both p = 0.001), respectively) and severity (7% and 27% (both p < 0.001), respectively). This study further observed that, when at least a 50% relief in physiological hot flashes was achieved, an improvement in secondary outcomes, such as quality of life parameters, including sleep, decreased fatigue, etc. was correspondingly noted. The authors proposed using this 50% threshold as a standard for evaluating other pharmacological and/or behavioural therapies.

Similar to the study of Loprinzi cited above (Loprinzi et al. 1998), positive results (38% reported at least a 50% decrease in HF frequency, and 63% reported a median decrease of 54% in the HF score) were noted in another uncontrolled pilot study among 23 androgen-deprived prostate cancer survivors using 12.5 mg venlafaxine b.i.d. (Quella et al. 1999).

Loprinzi’s group confirmed these findings in a larger double-blind, placebo-controlled study in 229 breast cancer survivors by investigating the dose-dependence of venlafaxine’s therapeutic effects (Loprinzi et al. 2000). After a baseline assessment week, venlafaxine treatments started at 37.5 mg daily and gradually increased to 75 mg or 150 mg daily for four weeks. Median HF frequencies and scores were reduced from baseline by 19 and 27% (placebo); 30 and 37% (37.5 mg); 46 and 61% (75 mg); and 58 and 61% (150 mg) (all p < 0.001). Adverse effects (dry mouth, decreased appetite, nausea, and constipation) were significantly higher compared to placebo in the 75 and 150 mg venlafaxine groups.

Three double-blind studies (Loibl et al. 2007; Buijs et al. 2009; Boekhout et al. 2011), previously cited in the clonidine section, compared various doses of venlafaxine and clonidine in breast cancer survivors; results from these studies were inconclusive viz. efficacy, but venlafaxine tended to have more AEs. And in another study that compared venlafaxine to gabapentin in 66 breast cancer survivors, venlafaxine showed similar efficacy but again more AEs (Bordeleau et al. 2010).

Contrasting results were found in one double-blind, placebo-controlled RCT in 120 prostate cancer survivors. Venlafaxine, at a dose of 75 mg/d, given either together with milk protein or with 160 mg/d soy isoflavones showed no significant effect of HF frequency or severity when compared to placebo plus milk protein or 160 mg/d soy isoflavones (Vitolins et al. 2013).

In conclusion, though some studies dispute venlafaxine’s efficacy (Evans et al. 2005; Vitolins et al. 2013) several other studies suggest a beneficial effect of venlafaxine in the treatment of post-menopausal vasomotor symptoms, also for breast and prostate cancer survivors, either alone (Loprinzi et al. 1998; Carpenter et al. 2007; Quella et al. 1999), or against placebo (Loprinzi et al. 2000), or compared to another active compound (Loprinzi et al. 2006; Loibl et al. 2007; Buijs et al. 2009; Boekhout et al. 2011; Bordeleau et al. 2010). However, the treatment duration of each of these studies was too short to investigate a potential increase in breast cancer recurrence. In addition to the AEs cited in these studies that have led to certain patients discontinuing the trial associated with venlafaxine (e.g., hypertension, decreased appetite, nausea/vomiting and constipation, sleeping problems, and sexual disturbances), breast enlargement is also a concern with this drug (Amsterdam et al. 1997) and is discussed below under *Safety of Antidepressants in Patients with a History of Breast Cancer*.

#### Desvenlafaxine

Desvenlafaxine (*O*-desmethylvenlafaxine) is the active metabolite of venlafaxine, and was studied in five large randomised, placebo-controlled trials (Speroff et al. 2008; Archer et al. 2009a; Archer et al. 2009b; Bouchard et al. 2012; Pinkerton et al. 2013) involving 2,582 post-menopausal patients for at least 12 weeks. All but one (Bouchard et al. 2012) of these studies showed a significant beneficial effect in treating vasomotor symptoms. However, none of these studies were performed in breast cancer survivors. Although the drug was approved as an antidepressant in the USA and Canada, the EMA did not approve desvenlafaxine for the treatment of major depression or menopausal complaints and the manufacturer therefore withdrew the European applications for both indications (EMEA 2014).

#### Mirtazapine, moclobemide and bupropion

Mirtazapine treatment (15–30 mg/d) for HF was serendipitously discovered in two depressed patients with post-menopausal complaints (Waldinger et al. 2000). Since HF and perspiration completely disappeared after one week of treatment, medication use was extended to two more patients without clinical signs of depression but with the same climacteric symptoms. The authors postulated that the 5-HT_2A_ blocking properties may account for the effect on HF. In a single-arm pilot study in 22 women (Perez et al. 2004), 59% with breast cancer of whom 9% and 45% were on raloxifene or tamoxifen treatments, respectively, patients received incremental doses of mirtazapine (7.5 (wash-in phase), 15 or 30 mg/day). At the end of the 4-week treatment, the median frequency and severity of HF decreased substantially by 52.5% and 59.5%, respectively. Similar results were obtained in another uncontrolled trial in 40 breast cancer survivors receiving 30 mg/d mirtazapine over 12 weeks (Biglia et al. 2007). A 55.6% reduction in HF frequency (p < 0.05) and 61.9% reduction in HF score relative to baseline (p < 0.05) were observed. Seven patients discontinued the study due to side effects, mostly somnolence. However, to date, no randomised clinical trials have been published and the benefit of this treatment cannot be adequately assessed.

There are also individual studies investigating two other anti-depressants, bupropion and moclobemide, in treating vasomotor symptoms. There have been anecdotal observations that bupropion, used for nicotine dependence and depression, can relieve HF symptoms. A pilot study lasting 4 treatment weeks was carried out in 21 patients, including breast and prostate cancer survivors, who built up to a 300 mg bupropion daily dose. Results showed that there was no significant reduction noted over that which would be expected from placebo (Perez et al. 2006).

Two different doses of moclobemide, 150 mg or 300 mg/d, were tested against placebo for 5 weeks in 30 post-menopausal women. The lower dose of this reversible, selective inhibitor of monoamine oxidase-A reduced the HF severity score by 69.8%, compared to 35.0% in the higher dose and 24.4% with placebo (Tarim et al. 2002).

### Safety of antidepressants in patients with a history of breast cancer

#### Prolactin and carcinogenesis

Certain antidepressant drugs modulate prolactin levels. Serum prolactin levels were investigated in 70 psychiatric patients where it was noted that patients on imipramine or amitriptyline treatment showed consistently higher prolactin levels compared to untreated controls (Turkington 1972). Prolactin is mitogenic, stimulates proliferation and suppresses apoptosis in breast and prostate cancer cells (Harvey et al. 2006) and is therefore important in the development of treatment-resistance in breast cancer cells (Carver et al. 2009).

Evidence from preclinical and clinical data show that elevated prolactin levels cause proliferation of breast tissue and result in breast enlargement, which may be markers for an increased risk of breast cancer development and important factors in the carcinogenicity of mammary tissue (Ingram et al. 1990; Arendt et al. 2011; Harvey et al. 2006; Clevenger et al. 2009; Carver et al. 2009). Breast enlargement was investigated and found in 39% of 59 women who received chronic SSRI or venlafaxine treatment for more than 8 weeks for depression (Amsterdam et al. 1997). Mammoplasia was reported in 64% of paroxetine-, 25% fluoxetine-, 25% sertraline- and 11% venlafaxine-treated patients. In the paroxetine group, a significant (p < 0.01) increase in prolactin serum concentrations was observed compared to pre-treatment values. Post-menopausal women with elevated plasma prolactin levels have a significantly higher risk of breast cancer (Hankinson et al. 1999). Several other reports on prolactin-related gynaecomastia, galactorrhoea, mastalgia or breast enlargement after tricyclic antidepressants, SSRIs and venlafaxine (Kropp et al. 2004; González et al. 2000; Bronzo & Stahl 1993; Bonin et al. 1994; Scurlock & Meehan 1996; Bonin et al. 1997), have confirmed these findings.

A mechanistic explanation of the prolactin increase after treatment with tricyclic antidepressants and SSRIs was provided by the finding that 5-HT neurons are believed to maintain a tonic inhibitory influence on dopamine function (Bonin et al. 1997). In addition to this indirect effect, tricyclic antidepressants and fluoxetine bind directly to intracellular, growth regulatory histamine receptors that are associated with anti-oestrogen binding sites. With this background in mind, the effects of amitriptyline and fluoxetine on tumour growth were investigated in rodents (Brandes et al. 1992), at concentrations corresponding to the treatment of human depression. Tumour latency decreased by 30-40% and frequency increased 2-fold in the DMBA (7,12-dimethylbenz[a]anthraene) model for chemically-induced breast cancer.

In contrast to this evidence, the carcinogenic risk of fluoxetine was evaluated in three carcinogenicity studies in rats and mice performed by the manufacturer (Eli Lilly & Co.). In these studies, fluoxetine was given over a period of 24 months in doses up to 10 mg/kg (Bendele et al. 1992), without any signs of treatment-related neoplasm development. However, in a different study in male rats, administration of 10 mg/kg i.p. fluoxetine did not affect resting serum prolactin levels but strongly potentiated stress-induced prolactin release (Krulich 1975).

#### Epidemiological evidence

To date, the clinical relevance of these effects in the safety assessment of antidepressant drugs has not been completely elucidated with regard to the risk of breast cancer development or recurrence. There is epidemiologic evidence that long-term use of the modern antidepressant drugs may be associated with a higher risk for developing breast cancer: In a case–control study using data from the Ontario Cancer Registry and controls, the risk for cancer development for long-term use of antidepressant drugs was investigated (Cotterchio et al. 2000). Compared with controls, use of tricyclic antidepressants for longer periods (>2 years) was associated with an elevated risk of breast cancer development (adjusted OR = 2.1, 95% CI 0.9-5.0), however, this increase in risk lacked statistical significance. Of the six most commonly reported antidepressant medications, only paroxetine use was associated with an increase in breast cancer risk (adjusted OR = 7.2, 95% Cl 0.9-58.3).

The Women’s Health Study (Kato et al. 2000) was performed in 15,270 women who participated in a mammographic screening programme. During an average of 7.3 years of follow-up, 566 incident cases of breast cancer were detected. The use of any type of psychotropic treatment at baseline was associated with a significantly increased relative risk of 1.39 (95% CI 1.11-1.74).

A follow-up case–control study using a larger sample from the same registry was performed by Steingart *et al*. (Steingart et al. 2003). In this study of 3,077 breast cancer patients, 441 used antidepressants; controls included 2,994 patients without breast cancer diagnosis, including 372 with antidepressant treatment. The analysis showed a significantly increased unadjusted risk for breast cancer for patient with ‘ever’ use of antidepressants (OR 1.17, 95% CI 1.01 – 1.36), especially for SSRI use (OR 1.33 (95% CI 1.07-1.66). Among the SSRIs, sertraline showed a significantly increased risk (OR 1.58 (95% CI 1.03-2.41) and paroxetine a borderline increased risk (OR 1.55, 95% CI 1.00-2.40). However, when risk was adjusted for other confounding factors associated with breast cancer risk, significance was lost, although the point estimates remained more or less the same.

In a large retrospective cohort study of 109,004 female health plan members who used various antidepressants between 1995 and 2000, paroxetine use was evaluated against breast cancer risk (Haque et al. 2005), where it was shown that the age-adjusted relative risk (RR) comparing “ever” users of paroxetine with other antidepressants was 1.12 (95% CI 0.96–1.31). Women who used paroxetine for 2 or more years did not show an increased risk of breast cancer compared to women who used it for a shorter period. Furthermore, use of SSRIs in general did not result in a statistically increased risk (RR 1.14 (95% CI 0.87-1.49)).

Results from another hospital-based case–control study (Kelly et al. 1999) in 5,814 women with breast cancer, 5,095 patients with other malignancies and 5,814 women without malignancies, researchers investigated the relative risk for developing breast cancer with regular use of antidepressants and structurally similar drugs. Though no significant increases in risk for any category of regular use were noted, the relative risk estimate for regular SSRI use in the previous year, 1.8, was of borderline statistical significance (95% confidence interval: 1.0, 3.3).

In a third case–control study, use of antidepressant drugs was investigated in patients with invasive breast cancer (n = 938) and controls (n = 771) (Moorman et al. 2003). Overall, women with invasive breast cancer did not report antidepressant use more frequently than controls (OR 1.0; 95% CI: 0.7-1.2). However, there was a trend that SSRI use for 36 months or longer was more prevalent in breast cancer patients than in controls (OR 2.2, 95% CI 0.8-6.3). Interestingly, carcinoma *in situ* cases reported antidepressant use significantly less frequently than controls (OR 0.6; 95% CI 0.4–0.8).

Another population-based case–control study included 975 elderly breast cancer cases and 1,007 age- and residence-matched controls conducted in Washington State (Chien et al. 2006). Antidepressant use information was obtained by structured in-person interviews. Overall, no association between ever use of antidepressants and breast cancer risk was noted (OR 1.2, 95% CI 0.9–1.6). However, compared to never users, ever SSRI users had significantly elevated risks of progesterone receptor (PR) negative and oestrogen receptor (ER) positive/PR-negative breast cancers (OR 1.8, 95% CI 1.1–3.6 and OR 2.0, 95% CI 1.1–3.8, respectively), but not of tumours with other hormone receptor profiles.

Contradicting results were obtained in a recent population-based study in 2,908 incident breast cancer cases and 2,927 control women (Wernli et al. 2009). There was no increased breast cancer risk in patients who received antidepressant drugs (OR 0.89, 95% CI 0.78-1.01).

In a large, retrospective cohort study based on prescription fillings by breast-cancer free women, (Wang et al. 2001), 38,273 females taking any antidepressant drug were compared to 32,949 women who took any other medication between 1989–1991. Use of antidepressant drugs was unrelated to the development of breast cancer (HR 1.04, 95% CI 0.87-1.25).

Coogan *et al*. (Coogan et al. 2008) used data from 820 invasive breast cancer cases in a case–control study and compared it to 2,852 hospitalised controls. The OR for all breast cancer cases was not elevated among regular users of SSRIs (OR 0.89, 95% CI 0.62-1.29). The results of this study were confirmed in 2,138 patients with invasive breast cancer and 2,858 controls (Coogan et al. 2005). The OR was 1.1 (95% CI 0.8-1.7) for regular use of SSRIs and 0.7 (95% CI 0.4-1.5) for use of 4 or more years. No ORs were elevated for any specifically-investigated SSRI.

Finally, this finding was again confirmed by a recent population-based case–control study in 2,129 women with primary invasive breast cancer and 21,297 randomly-selected control women (Ashbury et al. 2012). In this large study, no conclusive evidence of an increased breast cancer risk associated with the use of SSRIs was found, independent of the degree of serotonin reuptake inhibition or duration of use.

Discussion and Conclusion: Epidemiological studies, especially register-based ones, do rarely control for all of the possible confounding factors. Therefore, they do not prove a potential causal relationship. However, they may raise attention for possible associations and may motivate to perform a prospective study. Therefore, prospective long-term studies are needed to finally judge the risk of antidepressant treatment and breast cancer development.

#### Interaction with tamoxifen

Fluoxetine and paroxetine, and to a much lesser extent, possibly sertraline, citalopram and escitalopram, are inhibitors of the cytochrome P450 isoform CYP2D6 (Preskorn et al. 2007; Lam et al. 2002; Desmarais & Looper 2009; Desmarais & Looper 2010) that is important for metabolising tamoxifen, the therapy of choice in the adjuvant hormonal treatment of patients with oestrogen receptor-positive breast cancer. As a pro-drug, its active metabolite, endoxifen, is formed by a CYP2D6-mediated reaction (Pritchard 2010). SSRIs and, in particular, fluoxetine and paroxetine as the strongest CYP2D6 inhibitors, may therefore prevent the formation of the active metabolite from inactive tamoxifen (Crewe et al. 1997; Desta et al. 2004) and put breast cancer patients under anti-oestrogenic treatment at an increased risk of breast cancer recurrence (Singh et al. 2011). This was suggested by a population based cohort study in 2,430 women (Kelly et al. 2010), where the importance of CYP2D6 inhibition by SSRI could clearly be demonstrated. For paroxetine, the risk of death from breast cancer increased significantly with the proportion of time that tamoxifen was given concomitantly with paroxetine.

No increase in recurrence risk (OR 1.1, 95% CI 1.1-1.7) was demonstrated by a recent case–control study for citalopram and its S-isomer in 732 Danish patients who received tamoxifen for at least one year (Lash et al. 2011a; Lash et al. 2011b). This result may reflect the low inhibitory potency of citalopram and escitalopram. On the other hand, the lack of effect of citalopram or other SSRIs may be also due to the small number of patients who were studied in each of the subgroups.

In a recent matched case–control study in 3,901 breast cancer survivors (Goetz et al. 2013), patients were treated over 5 years either with tamoxifen alone, or after initial treatment with tamoxifen for 2 years, were switched to anastrozole, an aromatase inhibitor, which is neither a pro-drug nor metabolised by cytochrome CYP2D6. Homozygote-poor metabolisers for CYP2D6 tended to have a higher rate of breast cancer recurrence with continued tamoxifen use (OR 2.40; 95% CI 0.86-6.66, p = 0.09).

In a recent meta-analysis (Zeng et al. 2013), 20 clinical trials (11,701 breast cancer patients) were included where the impact of CYP2D6 polymorphisms on tamoxifen efficacy was assessed. Extensive metabolisers were associated with significantly improved disease-free survival (HR 1.37; 95% CI 1.12-1.69; p = 0.002) and overall survival (HR 1.25; 95% CI 1.03-1.50; p = 0.021).

On the basis of the available data, there is some evidence that co-administration of SSRIs (at least of fluoxetine and paroxetine) with tamoxifen may result in an increase in breast cancer recurrence in patients with anti-oestrogen therapy. Since alternative drugs are available that do not interact with CYP2D6, such combinations should be avoided (Binkhorst et al. 2013). In addition, pheno- or genotyping of patients for CYP2D6 poor metaboliser status may be warranted.

## Natural remedies and complementary medicine

In 2004, Fugate and Church assessed the efficacy and safety of non-hormonal, non-oestrogen treatments of menopause-associated vasomotor symptoms in a systematic review (Fugate & Church 2004). This review included non-prescriptional (dietary isoflavones, vitamin E, black cohosh, dong quai, evening primrose oil, physical activity, phytoestrogens, and red clover) as well as prescriptional treatments (clonidine hydrochloride, gabapentin, methyldopa, mirtazapine, propranolol hydrochloride, selective serotonin-reuptake inhibitors (SSRIs), and venlafaxine. However, in contrast to the present review, studies in cancer patients were explicitly excluded.

### Vitamin E

The efficacy of vitamin E in treating climacteric symptoms was investigated by Ziaei and colleagues using 400 IU/d softgel vitamin E tablets. They reported significantly reduced HF frequency (5.00 ± 3.34 vs. 3.19 ± 2.74) and severity scores (2.37 ± 0.74, 1.80 ± 0.87) when compared to placebo (p < 0.0001) (Ziaei et al. 2007).

The effects of vitamin E (800 IU daily) and placebo were studied over 4 weeks by Barton in a randomised, placebo-controlled, cross-over design in 120 breast cancer survivors. Although vitamin E preparations reduced the frequency of HF significantly compared to placebo in patients with a history of breast cancer and mild symptoms, the magnitude of the effect was small (one HF less per day), and therefore not clinically relevant (Barton et al. 1998).

### Phyto-oestrogens and vasomotor symptoms

The effects of phyto-oestrogens, plant-based compounds that exert oestrogen-like effects, on vasomotor menopausal symptoms were assessed in two Cochrane reviews in 2007 and 2013 (Lethaby et al. 2007; Lethaby et al. 2013). The more recent meta-analysis comprised 43 randomised controlled trials with a total of 4364 participants, however trials with breast cancer survivor had been excluded. Thirty-three of them have also been included in our review. Ten studies were excluded since they did not contain detailed information on HF frequency and/or severity or dealt with (mixed) dietary rather than pharmacological interventions. Certain trials found that some phyto-oestrogen treatments in peri- and post-menopausal women evoked a slight improvement in the frequency and severity of HF and night sweats when compared to placebo (Albertazzi et al. 1998; Scambia et al. 2000; Han et al. 2002; van de Weijer & Barentsen 2002; Jeri 2002; Sammartino et al. 2003; Nahas et al. 2004; Nahas et al. 2007; Khaodhiar et al. 2008; Cheng et al. 2007; Radhakrishnan et al. 2009; Ye et al. 2012; Aso et al. 2012; Mainini et al. 2013; D'Anna et al. 2007; D'Anna et al. 2009; Ferrari 2009; Evans et al. 2011) or with other compounds (Murkies et al. 1995; Crisafulli et al. 2004; Labos et al. 2013). However, many trials were small and confounded by a high risk of bias and unusually high placebo effect. Also, other investigations showed either no or an inconclusive benefit from soy isoflavone administration (Baber et al. 1999; Knight et al. 1999; Upmalis et al. 2000; St Germain et al. 2001; Burke et al. 2003; Faure et al. 2002; Campagnoli et al. 2005; Tice et al. 2003; Penotti et al. 2003; Secreto et al. 2004; Lewis et al. 2006). It is worth mentioning that the study that used the highest dose of isoflavones (200 mg/d) showed an exacerbation of HF symptoms (Levis et al. 2011). Those studies that compared phyto-oestrogens in breast and prostate cancer survivors likewise showed no conclusive evidence that phytooestrogen supplements effectively reduce HF (Quella et al. 2000; Van Patten et al. 2002; Nikander et al. 2003; MacGregor et al. 2005; Sharma et al. 2009). Under the heading, Phyto-oestrogens – Isoflavones (ISOF), Additional file 1 summarises the findings from the latest Cochrane review on this topic.

#### Isoflavones and breast/prostate cancer recurrence

With regard to preventing recurrence in breast cancer survivors, the data on beneficial effects of phyto-oestrogens are conflicting here as well. The inhibitory effect of enterolactone, a metabolite and marker of dietary lignans, on human aromatase by mammalian lignans and isoflavonoid phyto-oestrogens has been shown *in vitro* (Adlercreutz et al. 1993; Adlercreutz 1995), though the protective effects against breast cancer are only slight. It is not yet known whether this is due to a healthy diet or indeed evoked by the presence of dietary phyto-oestrogens (Adlercreutz 2002a; Adlercreutz 2002b). The effect of dietary phyto-oestrogen ingestion on the survival of breast cancer patients was investigated in 1,140 patients (Buck et al. 2011) by determining enterolactone for a median follow-up of 6.1 years. Enterolactone levels correlated positively with patient survival. The highest quartile of serum enterolactones was associated with a significantly reduced risk of death, but only in oestrogen receptor-negative tumours (HR 0.27 (95% CI 0.08-0.87)). It has been suggested that the high concentration of lignans in vegetarians, by inhibiting aromatase (=oestrogen synthetase) in peripheral and/or cancer cells and lowering oestrogen levels, may play a protective role as antipromotional compounds during growth of oestrogen-dependent cancers (Adlercreutz et al. 1993).

However, it is noteworthy that genistein, the most prevalent isoflavone in soy, can stimulate breast cancer growth and may interfere with the anti-tumour activity of tamoxifen (Duffy & Cyr 2003). Likewise, the isoflavone, biochanin A, may attenuate the effects of tamoxifen and the aromatase inhibitor, letrozole (Du et al. 2012; Singh et al. 2012; Ju et al. 2008).

### Melatonin

Chen *et al*. (Chen et al. 2014) studied the effect of melatonin at a dose of 3 mg/d over 16 weeks on HF frequency and severity in 95 breast cancer survivors in a double-blind placebo-controlled RCT. There was, compared to placebo, a significant improvement in subjects’ sleep quality, but no significant decrease in HF frequency or severity between treatments.

### Herbal supplements and vasomotor symptoms

Herbal supplements are widely used for vasomotor symptoms, with varying degrees of efficacy. Among the more frequently studied are hops (Heyerick et al. 2006; Erkkola et al. 2010), red clover (Hidalgo et al. 2005; Geller et al. 2009; Lipovac et al. 2012), flaxseed (Lewis et al. 2006; Colli et al. 2012; Pruthi et al. 2012), St. John’s Wort (*Hypericum perforatum*) (Al-Akoum et al. 2009; Abdali et al. 2010; Uebelhack et al. 2006; Briese et al. 2007), evening primrose (*Oenothera biennis*) (Farzaneh et al. 2013), French maritime pine bark (Pycngenol) (Yang et al. 2007; Kohama & Negami 2013); Sibiric Rhubarb (*Rheum rhaponticum*) (Heger et al. 2006; Kaszkin-Bettag et al. 2007; Kaszkin-Bettag et al. 2009; Hasper et al. 2009), valerian root (*Valeriana officinalis*) (Mirabi & Mojab 2013), Guaraná *(Paullinia cupana*) (Oliveira et al. 2013), and magnesium (Park et al. 2011). Summaries of these studies can be found in Additional file 1.

### Black cohosh (*Cimicifuga racemosa*)

One of the most widely studied and efficacious phytopharmaceuticals, CR (*Cimicifuga racemosa* L. *Actaea racemosa* L., black cohosh), is a perennial medicinal plant native to North America where it has been used for centuries in indigenous medicine for the treatment of many varied conditions. However, today’s sole accepted indications are menopause-related neurovegetative and emotional symptoms. Black cohosh or *Cimicifuga racemosa* extracts (CRE) are described in a 2003 monograph of the European Scientific Cooperative on Phytotherapy (ESCOP) as a pharmacologically-active treatment for climacteric symptoms (ESCOP Monographs 2003). Furthermore, in the 2010 community herbal monograph of the Committee on Herbal Medicinal Products (HMPC) of the EMA (EMEA 2010), a well-established use status was granted. CREs are registered as treatment for menopausal symptoms in many European countries (among them, Austria, Belgium, Czech Republic, Denmark, Finland, Germany, Hungary, Sweden, Switzerland, and UK) and Australia, South Korea, South Africa. In some countries (such as USA) CRE are used as a food supplement.

A recent Cochrane meta-analysis (Leach & Moore 2012), comprising 16 RCTs and recruiting a total of 2027 women with climacteric complaints, investigated CR for treating vasomotor symptoms. The authors point out that, due to the large degree of heterogeneity among the studies, pooling of the results was not possible. They state there is adequate justification for conducting more studies on this topic, but summarise that there was insufficient evidence to support the use of CR for menopausal symptoms. The conclusions of the authors, however, have been recently questioned by Beer *et al.* (Beer et al. 2013) who criticised that the selection of clinical studies in the Cochrane meta-analysis was biased. Not even half of the selected 16 studies were conducted within the indication using authorised products and several positive clinical studies were excluded or not identified (Osmers et al. 2005; Stoll 1987; Wuttke et al. 2003; Schellenberg et al. 2012). Using a re-analysis of all appropriate placebo-controlled clinical studies, they obtained a standardised mean difference of 0.385 in favour of CR (p < 0.0001).

Randomised, controlled trials that have shown a pharmacological effect and meeting the strict requirements of the FDA and EMA (FDA. U.S. Department of Health and Human Services Food and Drug Administration 2003; EMEA. Committee for Medicinal products for human use (CHMP) 2005), including at least 12 weeks duration, have been undertaken with CRE in 19 clinical studies among healthy menopausal women (Drewe et al. 2013; Lopatka et al. 2007; Vermes et al. 2005; Liske et al. 2002; Frei-Kleiner et al. 2005; Schellenberg et al. 2012; Osmers et al. 2005; Ross 2012; Newton et al. 2006; Geller et al. 2009; Stoll 1987; Wuttke et al. 2003; Nappi et al. 2005; Bai et al. 2007; Uebelhack et al. 2006; Briese et al. 2007; Oktem et al. 2007) and in breast cancer survivors (Hernández Munoz & Pluchino 2003; Rostock et al. 2011). Four other studies (Huang et al. 2013; Pockaj et al. 2004; Jacobson et al. 2001; Pockaj et al. 2006) did not meet the 12-week criterion, and can thus just be considered as supportive research, though for thoroughness, they are also cited in Additional file 1.

Various doses of ethanolic and isopropanolic formulations of CRE have been investigated in menopausal women (Drewe et al. 2013; Lopatka et al. 2007; Vermes et al. 2005; Liske et al. 2002) and results showed a significant decrease in HF symptoms and scores. When compared to placebo (Frei-Kleiner et al. 2005; Schellenberg et al. 2012; Osmers et al. 2005; Ross 2012), similarly positive results were observed. When combined or compared with other actives (Newton et al. 2006; Geller et al. 2009; Stoll 1987; Wuttke et al. 2003; Nappi et al. 2005; Bai et al. 2007; Uebelhack et al. 2006; Briese et al. 2007; Oktem et al. 2007; Huang et al. 2013), CRE was at least as efficacious as the other compound(s), except in (Newton et al. 2006) where herbal regimens did not reduce vasomotor symptoms.

Studies have also been conducted in breast cancer survivors using CRE alone (Pockaj et al. 2004), where HF were reduced by half; CRE vs. placebo (Jacobson et al. 2001; Pockaj et al. 2006) where no significant difference was achieved, and CRE with tamoxifen (Hernández Munoz & Pluchino 2003; Rostock et al. 2011), where again, significant improvements were noted.

#### Mechanism of action of CR

CR’s mechanism of action on climacteric symptoms is not yet clear. Selective modulation of oestrogen receptors, serotonergic, antioxidant and anti-inflammatory effects have been proposed (Ruhlen et al. 2008). CR binds to the serotonin receptors, 5-HT_1A_, 5-HT_1D_ and 5-HT_7_ (Burdette et al. 2003; Powell et al. 2008). From these, 5-HT_1A_ and 5-HT_7_ are also expressed in the hypothalamus and are involved in thermoregulation (Burdette et al. 2003; Hedlund et al. 2003; Naumenko et al. 2011).

There are conflicting data as to whether CREs act as phyto-oestrogens. Early studies in uterine and pituitary cells of ovariectomised rats showed that a chloroform fraction from a methanolic CRE did bind to oestrogen receptors (ER) (Jarry et al. 1985). Another study showed some evidence for an oestrogenic effect of CR in mice and oestrogen-dependent human breast cancer MCF-7 cells (Liu et al. 2001a). Partly contradicting these findings, the same group showed that CRE did not alter expression of different oestrogen-inducible genes (e.g., presenelin-2, progesterone receptor) (Liu et al. 2001b).

Later studies showed that a CRE did not bind to the oestrogen receptors Erα and ERβ (Jarry et al. 2003). Gene expression analysis showed that treatment with CREs down-regulates the expression of Erα (Gaube et al. 2007). The lack of an oestrogenic effect of CREs may be partly explained by an inhibition of the local oestrogen synthesis in breast tissue (Stute et al. 2007) or inhibition of the conversion of oestrone sulphate to active oestradiol in MCF-7 and MDA-123 breast cancer and human granulosa lutein cells (Rice et al. 2007). Furthermore, in MCF-7 cells, repetitive administration of CRE lead-induced gene expression opposite to 17β-oestradiol and more similar to tamoxifen (Gaube et al. 2007).

Two studies from the same group showed weak oestrogenic effects (e.g., lowering of LH secretion, bone remodelling, vaginal mucosa) in menopausal women and ovarectomised rats (Düker et al. 1991; Wuttke et al. 2006). However, most recent studies confirm the absence of any oestrogenic effect: In an oestrogen-sensitive fish (Japanese medaka), Zang *et al*. (Zhang et al. 2003) showed that, in contrast to the phyto-oestrogen genistein and oestradiol, CRE and some of its constituents (e.g., cimiracemoside A, 25-*O*-methyl-cimigenoside, actein, 26-deoxy-actein) did not change oestrogenic activity. In transcriptional-activation assays in yeast in oestrogen-dependent *S. cerevisiae* strain PL3, an isopropanolic extract (40%) did not show any oestrogenic activity (Pockaj et al. 2004).

Different herbal treatments, hormone therapy (HT) or placebo were administered to 351 patients in a 1-year, double-blind, placebo-controlled RCT (HALT-Study, Herbal Alternatives for Menopause (Reed et al. 2008)) study. After 12 and 52 weeks treatment, the HT group had a lower percentage of parabasal cells and vaginal dryness than did the placebo group (p < 0.05). Abnormal bleeding was reported in 16.9% of women. When used alone or as part of a multi-botanical product with or without soy dietary changes, CR exerted no effects on vaginal epithelium, endometrium, or reproductive hormones, indicating no local or systemic oestrogenic effects in patients.

CR’s lack of clinically-significant oestrogenic effects was further concluded by a lack of change in vaginal cytology and corresponding sexual hormones during 24-weeks of treatment in 152 peri- and post-menopausal women (Liske et al. 2002) where CRE treatment significantly reduced the frequency and severity of HF. These finding were corroborated by several other clinical studies, where the absence of systemic oestrogenic effects of CREs on sexual hormones and/or vaginal or endometrial thickness have been described (Nappi et al. 2005; Liske et al. 2002; Düker et al. 1991; Wuttke et al. 2006; Ruhlen et al. 2007; Rauš et al. 2006; Reed et al. 2008).

#### *In vitro* effects of CREs on cell proliferation in oestrogen-dependent breast cancer cells

The effect of CREs on human oestrogen receptor-positive and –negative breast cancer cell lines has been tested in different *in vitro* experiments where the results are often contradictory. The majority of published studies were able to show an inhibition of proliferation, or no effect, of CREs on cell proliferation.

The growth of oestrogen-dependent human breast cancer MCF-7 cells was not stimulated after a 48-hour treatment with an alcoholic CRE (Amato et al. 2002). An inhibitory effect on cell proliferation of oestrogen receptor-positive human mammary carcinoma cell lines was shown for the mammary carcinoma cell line 435 (Neßelhut et al. 1993), the human breast adenocarcinoma MCF-7 cells (Bodinet & Freudenstein 2002; Bodinet & Freudenstein 2004; Zierau et al. 2002; Garita-Hernandez et al. 2006; Al-Akoum et al. 2007; Rice et al. 2007; Gaube et al. 2007), T47D cells (Zava et al. 1998; Dixon-Shanies & Shaikh 1999), the ER^−^Her2 over-expressing breast cancer cell line MDA-MB-453 (Einbond et al. 2004; Einbond et al. 2006), EMT6 mouse mammary tumour cells (Rockwell et al. 2005) and MDA-MB-231 cells, which are associated with a highly-invasive potential (Al-Akoum et al. 2007; Hostanska et al. 2007; Rice et al. 2007).

CREs, or partly-isolated triterpene constituents of CREs, inhibited oestrogen-stimulated proliferation (Bodinet & Freudenstein 2004; Zierau et al. 2002; Al-Akoum et al. 2007) and enhanced the effects of tamoxifen (Bodinet & Freudenstein 2004; Al-Akoum et al. 2007), 5-fluorouracil, paclitaxel, doxorubicine and docetaxel (Rockwell et al. 2005; Einbond et al. 2006) in breast cancer cells.

This anti-cancer effect has been confirmed for some of the constituents of CR (Einbond et al. 2008). Among them, actein (β-D-xylopyranoside) showed an IC_50_ of 8.4 μM for the inhibition of growth of Her2 overexpressing MDA-MB-453 cells and of 32.5 μM for Her2 transfected and 45.8 μM for parenteral cells. Actein treatment altered the actin filament distribution of, and induced apoptosis in, these cells.

The mechanisms of the anti-proliferating effects have not yet been identified. However, modulation of cyclin D1 promoter activity and transcription activity of the p21 gene promoter may be involved (Garita-Hernandez et al. 2006). Changes in the gene expression pattern during chronic treatment with CRE have been investigated in MCF-7 cells in comparison with oestradiol and tamoxifen (Gaube et al. 2007). The pattern of gene induction was opposite to oestradiol and more similar to tamoxifen. Induced genes exhibited an antiproliferative and apoptosis-sensitising manner, as well as an increase in mRNAs coding for gene products involved in several stress response pathways.

Beyond those effects related to acute or sub-chronic administration, CRE may exert a chemopreventive effect. Treatment of rats starting from 56 weeks of age for 40 weeks resulted in a dose-dependent reduction of mammary adenocarcinomas (Einbond et al. 2012). This effect may be related to the observed reduced Ki-67 and cyclin D1 protein expression in fibroadenomas.

#### CR and tumour cell growth

It has been reported that inhibition of proliferation and induction of apoptosis are due to the action of triterpene glycosides and the cinnamic acid esters contained in CREs (Hostanska et al. 2004a; Hostanska et al. 2004b). In a further *in vitro* test for invasive potential of highly-invasive oestrogen receptor-negative MDA-MB 231 human breast cancer cells, 5 μg/ml doses of triterpene glycosides and the cinnamic acid esters reduced cell invasion by 34% and 25.5%, respectively (Hostanska et al. 2007). Similar results were obtained by Lupu *et al*. (Lupu et al. 2003). For various CREs (hexane, ethyl acetate and water), no oestrogenic activity (growth induction, regulation of oestrogen-dependent gene expression) has been demonstrated. In addition, anchorage-independent growth was investigated in oestrogen receptor positive MCF7 and T47D cells; thereby indicating possible progression of early stage breast cancer to a more aggressive state and the potential to build metastases (Mori et al. 2009). CR did not stimulate anchorage-dependent growth of breast cancer cells (Lupu et al. 2003).

The cytotoxic effects of powder from CR roots were shown in oestrogen-sensitive MCF-7 cells. Tamoxifen stimulated the growth of MCF-7 cells at high concentrations; on the other hand, when given alone, inhibited oestrogen-induced cell growth in a dose-dependent manner. Contrasting to these results, CR did not stimulate MCF-7 cell growth when given alone and blocked oestrogen-induced cell growth dose-dependently. The combination of tamoxifen with CR showed an enhanced (synergistic) cytotoxic effect of CR. It also inhibited growth of oestrogen-independent MDA-MB-231 breast cancer cells and this effect was synergistically enhanced by tamoxifen in a dose-dependent manner (Al-Akoum et al. 2007).

After acute administration of a CRE (at a dose of 6, 60, or 600 mg/kg) to mice, no signs of an oestrogenic effect could be detected (Einer-Jensen et al. 1996). Due to different oestrogenic effects of an isopropanolic CRE in various organs in ovariectomised rats, a selective oestrogen receptor modulator (SERM) activity has been postulated (Seidlová-Wuttke et al. 2003).

Stimulatory effects on tumour proliferation were studied in an *in vivo* oestrogen-receptor positive breast cancer model (Freudenstein et al. 2002), where mammary tumours were induced by 7,12-dimethylbenz[a]anthracene administration in female rats. After ovariectomy, growth of hormone-dependent mammary tumours was not stimulated by an isopropanolic CRE or placebo given over 6 weeks in contrast to animals treated with 450 μg/kg/day of the oestrogen, mestranol. Furthermore, there was neither a direct effect on uterine tissue proliferation nor an indirect effect on pituitary-secreted, oestrogen-regulated hormones exerted by the CRE.

The effect of CRE on *in vivo* tumour growth was further investigated in RUCA-I rats, an endometrial adenocarcinoma model. In contrast to tamoxifen, there was no stimulation of ectopic growth or an increase in the metastasising potential of the primary tumour noted with CR (Nisslein & Freudenstein 2004).

However, in contrast to the above investigations, one study using transgenic mice expressing c-erbB2 (MMTV-neu mouse model), showed that CRE significantly increased the incidence of lung metastases in tumour-positive animals when compared to mice fed a control diet free of isoflavones. Interestingly, no effect of CR on mammary tumour development was observed. This shows that CRE did not influence breast cancer risk if given prior to tumour formation (Davis et al. 2008). However, the literature indicates that these results have not been either confirmed nor refuted by other groups nor is supporting evidence available from clinical studies in patients with breast cancer.

#### CR and cytochrome P450 interaction

*In vitro* experiments using human liver microsomes suggested that methanolic extracts of CR significantly inhibit several cytochrome isoforms (IC_50_: CYP2B6: 49.2 μg/ml, CYP2C19: 23.9-36.3 μg/ml, and CYP2E1: 11.5 μg/ml) (Sevior et al. 2010). In a human interaction study, CR (80 mg) was given over 14 days to 19 healthy subjects. No clinically relevant interaction with CYP3A4 could be demonstrated using midazolam as the test drug (Gurley et al. 2006a). In MDA-MB-453 and MCF-7 human breast cancer cells, CYP1A1, CYP1B1 (Einbond et al. 2007; Gaube et al. 2007) and ABCC3 (MRP3) (Einbond et al. 2007) were up-regulated. One *in vitro* study showed that a CRE inhibited the formation of tamoxifen metabolites by CYP3A4 and CYP2D6, with IC_50_ values of 16.5 and 50.1 μg/mL, respectively. Eight triterpene glycosides were also identified as competitive CYP3A4 inhibitors, with IC_50_ values ranging from 2.3-5.1 μM, and protopine and allocryptopine alkaloids were shown to be competitive CYP2D6 inhibitors, with K_i_ values 78 and 122 nM, respectively (Li et al. 2011).

In 12 healthy volunteers, only a low, but clinically not relevant, interaction potential was shown for CYP2D6 when CR was given over 28 days (Gurley et al. 2005). The lack of an interaction of CR with CYP3A4, CYP2D6 and ABCB1 (P-glycoprotein) was confirmed in four separate human studies in healthy volunteers (Gurley et al. 2005; Gurley et al. 2006a; Gurley et al. 2008; Gurley et al. 2006b).

Although there is evidence for an interaction potential of CR and its constituents with tamoxifen metabolism, the clinical data nevertheless indicate that there is no relevant inhibition of CYP2A4 or CYP2D6. Therefore, a clinically relevant interaction of CR with tamoxifen is most unlikely.

#### Clinical effects of CR with regard to tumour development

Three notable studies assess breast density measurements as a biomarker for the risk of breast cancer development.

In a large prospective, open study in 400 post-menopausal women, the endometrial safety and breast density were studied before and after a 52-week CRE treatment (Rauš et al. 2006). No case of hyperplasia occurred and no serious adverse endometrial outcomes were found, indicating endometrial safety. Endometrial thickness, measured by endovaginal ultrasonography, did not increase during treatment. An increase in breast density was found in only one woman who was diagnosed with invasive breast cancer which, based on history, was unrelated to the CR.

In the second trial, the influence of CR on mammary breast density was investigated in a prospective, open, uncontrolled safety study in 74 post-menopausal women (Hirschberg et al. 2007). Breast density was assessed by mammography and proliferation of breast tissue by fine needle aspiration histology using Ki-67/MIB-1 monoclonal antibody. Assessment performed at baseline and after 24 weeks showed no increase in mammographic breast density or breast cell proliferation.

Lundström confirmed these results by comparing two studies (Lundström et al. 2011): The first, a prospective, open, uncontrolled drug safety study in 65 post-menopausal women who were treated with 20 mg CR twice daily and the second, a randomised, placebo-controlled clinical study in 154 post-menopausal women who were treated with either oestradiol 2 mg/norethisterone acetate 1 mg (E2/NETA), tibolone 2.5 mg or placebo. Mammograms were performed at baseline and showed comparable breast density for each treatment. Renewed mammograms after 24 weeks of treatment showed that both E2/NETA and tibolone significantly increased breast density (14.3%, and 2.3%, respectively, both p < 0.001) while CR and placebo had no effect on breast density. These differences were highly significant (p < 0.0001) (Lundström et al. 2011).

In a study evaluating 149 patients, two doses (39 and 127.3 mg/d) of an isopropanolic CRE were studied in a double-blind RCT for the treatment of climacteric symptoms over a 24-week period (Liske et al. 2002). Compared to baseline, both treatments significantly decreased climacteric symptoms, but no oestrogen-induced change in vaginal mucosal thickness or sex hormones were observed, indicating a lack of local and overall oestrogenic effects.

In a retrospective case–control study (Rebbeck et al. 2007), use of CRE was associated with a significantly lower risk of developing breast cancer (adjusted OR 0.39; 95% CI 0.22-0.70), however, the sub-sample of patients treated with CR was rather small. Similar results were found in a large German case–control study in 10,121 post-menopausal women (Obi et al. 2009), where 3,464 incident breast cancer cases were compared to 6,657 controls. Ever use of isopropanolic CRE was associated with a borderline reduced risk for the development of breast cancer (OR 0.80; 95% CI 0.63-1.00). No protective effect of CR (HR 1.17; 95% CI 0.75-1.82) was seen in a large case–control study (Brasky et al. 2010) in 35,016 post-menopausal women, however, the number of incident breast-cancer cases in the CR sub-population was too small (n = 21) to allow a meaningful conclusion.

The risk of breast cancer recurrence where the primary outcome was disease-free survival was investigated in 18,861 patients having a previous breast cancer diagnosis (Heinecke-von Zepelin et al. 2007), among whom 1,102 patients received isopropanolic CRE for a mean overall observation period of 3.6 years. Controlling for age, tamoxifen use and other confounders, the Cox regression model demonstrated a statistically significant protective effect of isopropanolic CR on recurrence rate (HR 0.83, 95% CI 0.69-0.99).

#### Males

Several studies have shown that those anti-androgen treated, or surgically castrated, men who develop severe HF symptoms respond well to treatments that are effective against menopausal symptoms in women, in particular, oestrogens, though often accompanied with breast tenderness, gynaecomastia and an increased risk of cardiovascular and thrombembolic events (Adelson et al. 2005).

Alternatively, *in vitro* experiments indicate that treatment with CREs may benefit prostate cancer patients. Anti-proliferative effects of CR for several prostate cancer cell lines and *in vivo* tumours have been observed. Most studies describe induction of apoptotic cellular response and subsequent reduction in proliferation and partly prostate-specific antigen secretion in androgen dependent tumour cells (LNCaP) (Hostanska et al. 2005; Jarry et al. 2007; Jarry et al. 2005). In addition, 5α-reductase (the key enzyme for dihydro-testosterone synthesis) was inhibited by CR in the rat prostate (Seidlová-Wuttke et al. 2006), possibly indicating its suitability in preventing and treating prostate cancer and benign prostate hyperplasia.

Further, one study showed *in vivo* anti-proliferative and growth inhibitory effects for implanted prostate cancer in immunodeficient (nu/nu) athymic nude mice (Seidlova-Wuttke et al. 2006). However, a controlled clinical trial is yet to be performed.

## Discussion

The most effective treatment of climacteric symptoms is HT with oestrogen or a combination of oestrogen and progestins (MacLennan et al. 2009). However, the benefits are partly outweighed by a significantly increased risk for the development of breast cancer (Collaborative Group on Hormonal Factors in Breast Cancer 1997; Porch et al. 2002; Rossouw et al. 2002; Weiss et al. 2002; Beral et al. 2002; Beral & Million Women Study Collaborators 2003; Chlebowski et al. 2009). After publication of the WHI studies (Rossouw et al. 2002) and the Million Women Study (Beral & Million Women Study Collaborators 2003), HT use decreased drastically worldwide (Hersh et al. 2004; Canfell et al. 2008; Antoine et al. 2011), accompanied by a significant decrease in breast cancer incidence (Canfell et al. 2008; Ravdin et al. 2007; Canfell et al. 2009).

Recently, the risk-benefit ratio of HT was reassessed for various ages and time intervals since menopause onset. The North American Menopause Society (NAMS) (NAMS 2012) proposed that duration of treatment should be limited to younger women up to the age of 50 to 59 years; beyond which, HT is associated with increased risks. Similar recommendations were issued by the International Menopause Society (IMS) in 2011 (Sturdee et al. 2011), which considered HT as a first-line therapy choice for climacteric symptoms. Finally, a global consensus on the use of HT was obtained by The American Society for Reproductive Medicine, The Asia Pacific Menopause Federation, The Endocrine Society, The European Menopause and Andropause Society, The International Menopause Society, The International Osteoporosis Foundation and The North American Menopause Society (de Villiers et al. 2013). They stated that HT “is the most effective treatment for vasomotor symptoms associated with menopause at any age, but benefits are more likely to outweigh risks for symptomatic women before the age of 60 years or within 10 years after menopause”. Although some HT restrictions have been withdrawn, there is still a considerable need for non-hormonal treatment alternatives, especially for elderly post-menopausal women or cancer patients of both genders. Therefore, this present work aimed to assess the risk-benefit ratio of various non-hormonal treatment options. Among them, four non-hormonal treatments appear to have significant evidence for a beneficial effect in treating vasomotor climacteric or androgen-ablation symptoms, although some studied the effects over a period shorter than 12 weeks: gabapentin/pregabalin (Loprinzi et al. 2002a; Guttuso et al. 2003; Reddy et al. 2006; Loprinzi et al. 2007; Butt et al. 2008; Saadati et al. 2013; Agarwal et al. 2014; Pinkerton et al. 2014; Loprinzi et al. 2010; Pandya et al. 2004; Pandya et al. 2005; Bordeleau et al. 2010; Loprinzi et al. 2009; Moraska et al. 2010), SSRIs (Stearns et al. 2003; Stearns et al. 2005; Simon et al. 2013; Huang et al. 2013; Stearns et al. 2000; Gordon et al. 2006; Grady et al. 2007; Kerwin et al. 2007; Aedo et al. 2011; Kimmick et al. 2006; Wu et al. 2009; Suvanto-Luukkonen et al. 2005; Oktem et al. 2007; Loprinzi et al. 2002b; Barton et al. 2010; Barton et al. 2003; Defronzo Dobkin et al. 2009; Freedman et al. 2011; Freeman et al. 2011; Carpenter et al. 2012; Ensrud et al. 2012), venlafaxine/desvenlafaxine (Evans et al. 2005; Loprinzi et al. 2006; Loprinzi et al. 1998; Carpenter et al. 2007; Quella et al. 1999; Loprinzi et al. 2000; Loibl et al. 2007; Buijs et al. 2009; Boekhout et al. 2011; Bordeleau et al. 2010; Vitolins et al. 2013; Speroff et al. 2008; Archer et al. 2009a; Archer et al. 2009b; Cheng et al. 2013; Bouchard et al. 2012; Pinkerton et al. 2013), and CREs (Drewe et al. 2013; Lopatka et al. 2007; Vermes et al. 2005; Liske et al. 2002; Frei-Kleiner et al. 2005; Schellenberg et al. 2012; Osmers et al. 2005; Ross 2012; Newton et al. 2006; Geller et al. 2009; Stoll 1987; Wuttke et al. 2003; Nappi et al. 2005; Bai et al. 2007; Uebelhack et al. 2006; Briese et al. 2007; Oktem et al. 2007; Huang et al. 2013; Pockaj et al. 2004; Jacobson et al. 2001; Pockaj et al. 2006; Hernández Munoz & Pluchino 2003; Rostock et al. 2011).

Randomised, controlled trials that have shown a pharmacological effect and are at least 12 weeks duration, as required by FDA and EMA (FDA. U.S. Department of Health and Human Services Food and Drug Administration 2003; EMEA. Committee for Medicinal products for human use (CHMP) 2005) have only been undertaken for gabapentin (Guttuso et al. 2003; Reddy et al. 2006); (Saadati et al. 2013) #3447 (Agarwal et al. 2014; Pinkerton et al. 2014); SSRIs: (Simon et al. 2013; Aedo et al. 2011; Suvanto-Luukkonen et al. 2005; Oktem et al. 2007), venlafaxine (Evans et al. 2005; Boekhout et al. 2011; Vitolins et al. 2013), desvenlafaxine (Speroff et al. 2008; Archer et al. 2009a; Archer et al. 2009b; Cheng et al. 2013; Bouchard et al. 2012; Pinkerton et al. 2013), isoflavones (Albertazzi et al. 1998; Han et al. 2002; van de Weijer & Barentsen 2002; Jeri 2002; Sammartino et al. 2003; Nahas et al. 2004; Nahas et al. 2007; Khaodhiar et al. 2008; Cheng et al. 2007; Radhakrishnan et al. 2009; Ye et al. 2012; Aso et al. 2012; Mainini et al. 2013; D'Anna et al. 2007; D'Anna et al. 2009; Ferrari 2009; Evans et al. 2011; Murkies et al. 1995; Crisafulli et al. 2004; Labos et al. 2013; Upmalis et al. 2000; Faure et al. 2002); hops (Heyerick et al. 2006); red clover (Hidalgo et al. 2005; Lipovac et al. 2012), flaxseed (Colli et al. 2012), St. John’s wort (Uebelhack et al. 2006; Briese et al. 2007), French maritime pine bark (Yang et al. 2007; Kohama & Negami 2013), Sibiric Rhubarb (Heger et al. 2006; Kaszkin-Bettag et al. 2007; Kaszkin-Bettag et al. 2009; Hasper et al. 2009), and CREs (Drewe et al. 2013; Lopatka et al. 2007; Vermes et al. 2005; Liske et al. 2002; Frei-Kleiner et al. 2005; Schellenberg et al. 2012; Osmers et al. 2005; Ross 2012; Newton et al. 2006; Geller et al. 2009; Stoll 1987; Wuttke et al. 2003; Nappi et al. 2005; Bai et al. 2007; Uebelhack et al. 2006; Briese et al. 2007; Oktem et al. 2007; Hernández Munoz & Pluchino 2003; Rostock et al. 2011).

## Conclusion

Several non-hormonal alternatives to hormonal therapy have been established and confirmed for the treatment of vasomotor climacteric symptoms in peri- and post-menopausal women. Although there are indications that these treatments are useful in patients with a history of breast cancer, this still requires confirmation by larger clinical trials.

This systematic analysis did not carry out any of its own clinical research involving patients and the statements relating to the ethical standards laid down in the 1964 Declaration of Helsinki and its later amendments thus do not apply.

## Additional file

Additional file 1:
**Clinical effect of non-hormonal treatments in menopausal and cancer patients.**
