# Supplementary material for: A systematic review of non-hormonal treatments of vasomotor symptoms in climacteric and cancer patients
Source: Springerplus. 2015 Feb 10;4:65. doi: 10.1186/s40064-015-0808-y (PMC4331402; doi:10.1186/s40064-015-0808-y)
Supplement: Additional file 1: — Clinical effect of non-hormonal treatments in menopausal and cancer patients. [file 40064_2015_808_MOESM1_ESM.doc]

**Additional file 1: Clinical effect of non-hormonal treatments in menopausal and cancer patients**

| **Author, Year**  **Reference** | **N ^1)^** | | **Study Type** | | **Design** | | **Patient Population & Treatment**  (all medication is p.o. unless otherwise specified) | | | | | | | **Duration** | | | **Primary Outcome** | | | | **Results** |
| --- | --- | --- | --- | --- | --- | --- | --- | --- | --- | --- | --- | --- | --- | --- | --- | --- | --- | --- | --- | --- | --- |
| **Dopamine agonists** | | | | | | | | | | | | | | | | | | | | | |
| Nesheim, 1981 | 40 | | RCT | | Double-blind, PLA-controlled, cross-over | | Post-menopausal women  Methyldopa 500-1000 mg/d vs. PLA | | | | | | | 30 days | | | HF frequency | | | | Number of HF decreased significantly (p=0.004) by 38% (PLA) and 65% methyldopa |
| Andersen, 1986 | 40 | | RCT | | Double-blind, PLA-controlled, cross-over | | Post-menopausal women  titrated up to 1125 mg/d α-methyldopa | | | | | | | 4 weeks | | | HF frequency and severity | | | | α--methyldopa decreased HF severity (p<0.05), however, frequency was only borderline affected (p>0.05). |
| Zichella, 1986 | 75 | | RCT | | PLA controlled | | Post-menopausal women:  Bromocriptine 3.75 mg/d. vs. Liposom 40 mg/d i.m. vs. veralipride 100 mg/d vs. domperidone 10 mg/d vs. PLA | | | | | | | 20 days | | | HF frequency, severity | | | | Active treatments were significantly (66-75% vs. baseline, p<0.001 for all) more effective than PLA (20%, p<0.05). |
| **Adrenergic Agonists/Clonidine (CD)** | | | | | | | | | | | | | | | | | | | | | |
| Chow, 1993 | 30 | | RCT | Parallel group, *verum* vs. no treatment | | | | Post-menopausal women with on average 44.9 HF per week  0.025 up to 0.075 mg CD b.i.d vs. no treatment | | | | 4 weeks | | | | | | HF frequency, severity, duration | | | Significant improvement in the frequency, severity and duration of HF |
| Nagamani, 1987 | 29 | | RCT | Prospective, double-blind PLA-controlled | | | | Post-menopausal women with 10 to 100 HF per week  **transdermal** administration  corresponding to 0.1 mg/d CD vs. PLA | | | | 8 weeks | | | | | | HF frequency, severity, duration | | | Significant improvements in HF frequency (-80%, p<0.04), severity (73%, p<0.04) and duration (67%, p<0.03) when compared to placebo (36%, 29% and 21%), respectively. |
| Clayden, 1974 | 100 | | RCT | Double-blind PLA-controlled, cross-over | | | | Post-menopausal women with on average 44.9 to 50.2 HF per week  0.025 up to 0.075 mg CD b.i.d. vs. PLA | | | | 4 weeks of each treatment | | | | | | HF frequency, severity, duration | | | Significant improvements in the frequency, severity and duration of HF |
| Edington, 1980 | 66 | | RCT | Double-blind, PLA- controlled, cross-over  (four separate trials) | | | | Menopausal women with ≥ 30 HF per week  0.050 mg CD b.i.d. vs. PLA | | | | 4 weeks | | | | | | HF frequency, severity, duration | | | Overall, frequency, severity and duration of attacks were reduced by CD in 78-89%, and by PLA in 50-53% of the patients. More AEs in the CD group. |
| Laufer, 1982 | 10 | | unknown | Single-blind, dose-response | | | | Post-menopausal women with on average 0.84 HF per day  PLA, 0.1, 0.2, and 0.4 mg CD | | | | 2 weeks of each treatment: 8 weeks total | | | | | | HF frequency, severity, duration | | | Maximum dose (0.4 mg) significantly reduced the HF frequency by 46% (p<0.05%). More AEs in CD group. |
| Wren, 1986 | 19 | | RCT | Double-blind PLA-controlled, cross-over | | | | Post-menopausal women with on average 8.6 to 13.1 HF per day  0.05 mg CD b.i.d. vs. PLA | | | | 4 weeks | | | | | | HF frequency, | | | No statistical reduction in number of HF |
|  |  | |  |  | | | | |  | | | |  | | | | | |  | |  |
| Goldberg, 1994 | 110 | | RCT | Double-blind PLA-controlled, cross-over | | | | **Breast cancer survivors** receiving tamoxifen with ≥ 7 HF per week:  CD transdermal equivalent to 0.1 mg/d vs. PLA | | | | 4 weeks | | | | | | HF frequency, severity | | | Reduction in frequency (20%; p<0.0001) and severity (10%, p=0.02) with CD. Significant AE mouth dryness, constipation, itchiness under patch, and drowsiness. |
| Loprinzi, 1994 | 70 | | RCT | Double-blind, cross-over | | | | **Prostate cancer survivors**  after orchiectomy with ≥ 7 HF per week  0.1 mg/d transdermal clonidine | | | | 4 weeks | | | | | | HF frequency, severity | | | Transdermal CD did not significantly decrease (p=0.57) HF frequency or severity |
| Pandya, 2000 | 194 | | RCT | Double-blind, PLA-controlled | | | | **Breast cancer survivors** receiving tamoxifen with on average 7.4 to 8.0 HF per day;  0.1 mg/d CD vs. PLA | | | | 8 weeks | | | | | | Frequency,  duration, and severity of HF | | | Reduction in HF frequency was greater in the CD group than PLA at 4 (37% vs. 20%, p=0.001) and 8 weeks (38% vs. 24%, p=0.006). Patients receiving CD reported more difficulty sleeping (41% vs. with 21%; p=0.02). |
| Loibl, 2007 | 80 | | RCT | Double-blind, Phase III | | | | | **Breast cancer survivors** with ≥ 14 HF per week  0.075 mg CD b.i.d. vs. 37.5 mg VEN b.i.d. | | | 4 weeks | | | | | | | HF frequency | | VEN is significantly (p=0.025) more effective than CD in reducing the frequency of HF. Significantly (p=0.05) more nausea in the VEN group. |
| Buijs, 2009 | 60 | | RCT | Double-blind, cross-over | | | | | **Breast cancer survivors** with ≥ 14 HF per week  75 mg/d VEN vs. 0.05 mg CD b.i.d. | | | 8 weeks | | | | | | | HF frequency and severity | | Median reduction in HF score was comparable, 49% for VEN and 55% for CD. Dropouts due to toxicity: 14/59 VEN; 5/53 CD (p=0.038) |
| Boekhout, 2011 | 102 | | RCT | Double-blind, PLA-controlled | | | | **Breast cancer survivors** with ≥ 2 HF per day  75 mg/d VEN vs.  0.1 mg/d CD vs. PLA | | | | 12 weeks | | | | | | HF score | | | Overall, during the 12 weeks, HF scores were significantly lower in the CD and VEN group vs. PLA (p<0.001 and p=0.045, respectively); but when compared to PLA at week 12 specifically, only CD showed lower HF scores, p=0.03; VEN p=0.07. Nausea (p=0.02), constipation (p=0.04) higher with VEN |
| **Gabapentin (GP) / Pregabalin (PG)** | | | | | | | | | | | | | | | | | | | | | |
| Loprinzi, 2002 | 24 | | OBS | | | Uncontrolled | | | | Post-menopausal women, doses increasing from 300 to 900 mg/d GP | | | | | 4 weeks | | | | | HF frequency and score (frequency times avg. severity) | Reduced HF frequency (66%) and severity score (70%) compared to baseline. |
| Guttuso, 2003 | 59 | | RCT | | | Double-blind, PLA-controlled | | | | Post-menopausal women with ≥ 7 HF per day  900 mg/d GP vs, PLA (12 weeks) followed by a 5-week open-label treatment with 900 to 2700 mg/d | | | | | 12 week /  5 weeks | | | | | HF frequency and severity | At week 12, HF frequency and score decreased with GP by 45% (p=0.02) and 54% (p=0.01) vs. 29% and 31% with PLA, respectively. Common AEs in GP group: somnolence, dizziness, rash, peripheral oedema. |
| Reddy, 2006 | 60 | | RCT | | | Double-blind, PLA-controlled | | | | Post-menopausal women with ≥ 50 HF per week  2400 mg/d GP, 0.625 mg/d oestrogen or PLA | | | | | 12 weeks | | | | | HF frequency and severity | At week 12, similar reductions in the HF composite score for both oestrogen (72%, p=0.016) and GP (71%, p=0.004); both greater than the reduction seen with PLA (54%). More AEs with GP: headache, dizziness, disorientation. |
| Loprinzi, 2007 | 118 | | RCT | | | Double-blind,  PLA-controlled | | | | Menopausal women with HF insufficiently controlled by AD alone with ≥ 14 HF per weekGP (300 to 900 mg/d) + AD versus GP alone | | | | | 5 weeks | | | | | HF frequency | Both with and without the AD, GP evoked an approximately 50% median reduction in HF frequency |
| Butt, 2008 | 200 | | RCT | | | Double-blind, PLA-controlled | | | | Post-menopausal women with ≥ 14 HF per week  300 mg/d GP t.i.d. vs. PLA | | | | | 4 weeks | | | | | HF frequency and severity | HF scores and frequency decreased by 51.0% and 45.7% (GP) compared with 26.5% and 24.7% (PLA) (both p<0.001). |
| Saadati, 2013 | 60 | | RCT | | | PLA-controlled | | | | Post-menopausal women with on average 13.1 HF per week  300 mg/d GP t.i.d. vs. PLA | | | | | 12 weeks | | | | | HF frequency and severity | Significant difference at 12 weeks between GP and PLA groups with regard to HF frequency and severity (both p<0.001) |
| Agarwal, 2014 | 50 | | RCT | | | Double-blind, PLA-controlled | | | | Post-menopausal women with on average 6 to 7.1 HF per day  900 mg/d GP vs. PLA | | | | | 24 weeks | | | | | HF frequency and severity | Compared to PLA, GP reduced HF frequency by 59.1% (p=0.008) at 12 weeks and 60.6% (p=0.005) at 24 weeks; and reduced the composite score by about 80% at 12 and 24 weeks (both p=0.001); |
| Pinkerton, 2014 | 600 | | RCT | | | PLA-controlled | | | | Peri- and post-menopausal women with ≥ 7 HF per day  Gastroretentive GP 600 mg AM/1200 mg PM vs. PLA | | | | | 12 weeks 24 weeks for secondary endpoints only | | | | | HF frequency and severity | Although statistically significant (HF frequency: p=0.0007, HF severity: p=0.012), gastroretentive GP was only slightly better than PLA |
| Loprinzi, 2010 | 207 | | RCT | | | Double-blind, PLA-controlled | | | | Post-menopausal women with 4-9 and ≥ 10 HF per day  2 x 75 mg/d PG vs. 2 x 150 mg/d PG vs. PLA | | | | | 6 weeks | | | | | HF frequency and severity | HF score decreased by 50% (PLA), 65% (2x75 mg PG), and 71% (2x150 mg PG), resp. (p=0.009 and p=0.007 versus PLA arm). |
|  |  | |  | | |  | | | |  | | | | |  | | | | |  |  |
| Pandya, 2004 | 22 | | OBS | | | Uncontrolled | | | | **Breast cancer survivors on tamoxifen** with ≥ 2 HF per day  3 x 300 mg/d GP | | | | | 4 weeks | | | | | HF duration, frequency and severity | Reduced HF duration 73.6% (p=0.027), frequency (44.2%, p<0.001) and severity (52.6%, p<0.001) compared to baseline. 4 drop-outs due to AEs; 8/16 women completing the study showed a complete response. |
| Pandya, 2005 | 420 | | RCT | | | Double-blind, randomised, PLA-controlled | | | | **Breast cancer survivors** with on average ≥ 2 HF per day  300 or 900 mg/d GP vs. PLA | | | | | 8 weeks | | | | | HF frequency and severity | At 8 weeks, only the 900 mg showed a significant reduction in HF frequency (44%, p<0.0001) and severity (46%; p<0.0001) versus 15% and 15% in the PLA group |
| Bordeleau, 2010 | 66 | | RCT | | | Group-sequential, open-label, cross-over trial | | | | **Breast cancer survivors** with ≥ 14 HF per week  37.5 mg/75 mg VEN vs. 300, 600, 900 mg/d GP | | | | | 4 weeks | | | | | HF score | Both agents reduced HF score by about 66% (p<0.001). 32% preferred GP, 68% VEN. VEN showed more nausea, appetite loss, constipation, and reduced negative mood changes than GP, GP had more dizziness and appetite compared with VEN (all p<0.05) |
| Loprinzi, 2009 | 223 | | RCT | | | Double-blind, PLA-controlled | | | | **Prostate cancer** **survivors** with ≥ 14 HF per week  300, 600 and 900 mg/d GP vs. PLA | | | | | 4 weeks | | | | | HF frequency and severity | Slight to moderate decrease in HF frequency and severity over PLA that reached significance only in the 900 mg group (p=0.02). |
| Moraska, 2010 | 147 | | Uncon-trolled | | | Open-labeled continuation of RCT in | | | | **Prostate cancer** **survivors** with ≥ 14 HF per week  Titration up to 900 mg/d GP | | | | | 8 weeks | | | | | HF frequency | Moderate reduction in HF frequency maintained |
| **Paroxetine (PAR)** | | | | | | | | | | | | | | | | | | | | | |
| Stearns, 2003 | 165 | | RCT | | Double-blind, PLA-controlled, parallel groups | | | | | Post-menopausal women with ≥ 2 HF per day,  one week PLA run-in, then  PLA or 12.5 or 25 mg/d PAR SR for 6 weeks | | | | | 6 weeks | | | | | HF frequency and composite score | Significant median reductions in HF frequency and composite score (vs. PLA): 12.5 mg: 62.2% and 3.3 (p=0.007); 25 mg: 64.6% and 3.2 (p=0.03) compared to 37.8% and 1.8 (PLA) |
| Stearns, 2005 | 151 | | RCT | | Double-blind, cross-over, PLA-controlled | | | | | Post-menopausal women with ≥ 2 HF per day,  10 or 20 mg/d PAR, followed or preceded by 4 weeks PLA | | | | | 9 weeks | | | | | HF frequency and severity | HF frequency and score by 40.6% and 45.6% vs. 13.7% and 13.7% with PLA (p=0.0006 and p=0.0008, resp.), and 20 mg PAR by 51.7% and 56.1% vs. 26.6% and 28.8% with PLA (p=0.002 and p=0.004, resp.) |
| Simon, 2013 | 1184 | | 2 RCTs | | Double-blind, PLA-controlled, Phase III (both) | | | | | Post-menopausal women with ≥ 7 HF per day  7.5 mg/d PAR vs. PLA | | | | | 12 and 24 weeks | | | | | HF frequency and severity | In both studies, PAR decreased HF frequency and partly severity at the end of treatment: 12-week study (p=0.009 and p=0.2893); 24-week study (p=0.0001 and p=0.0114). |
| Huang, 2013 | 120 | | RCT | | Active controlled | | | | | Peri-menopausal women  20 mg/d PAR vs. 20 mg/d PAR + 40 mg/d isopropanolic CRE *) | | | | | 8 weeks | | | | | HF frequency and severity (Kupperman index) and depression (HAMD) | In both studies, PAR decreased HF frequency and severity at the end of treatment: 9.89 ± 3.76 but not as well as the combined treatment 15.75 ± 5.84 (P<0.01). Likewise, the combined treatment was superior to PAR alone for the HAMD 88.3% vs, 78.3% (p<0.05) |
|  |  | |  | |  | | | | |  | | | | |  | | | | |  |  |
| Stearns, 2000 | 30 | | OBS | | Observational, uncontrolled | | | | | **Breast cancer survivors** with ≥ 2 HF per day,  PAR 10 mg/d for 1 week followed by 20 mg/d for 4 weeks | | | | | 6 weeks (1 week baseline, 5 treatment) | | | | | HF frequency and severity score | Mean reduction of 67% (95% CI: 56%-79%) for frequency and 75% (95% CI: 66%-85%) for severity score |
| **Sertraline (SERT)** | | | | | | | | | | | | | | | | | | | | | |
| Gordon, 2006 | 102 | | RCT | | Double-blind, PLA-controlled, cross-over | | | | | | Menopausal women with on average 45.4 to 49 HF per week  SERT 50 mg/d or PLA | | | | | 4 weeks | | | | HF frequency, severity score | Compared to PLA, significantly fewer HF (p=0.002), similar severity and significantly decreased HF score (p=0.001) |
| Grady, 2007 | 99 | | RCT | | Blinded, parallel, PLA-controlled | | | | | | Peri- and post-menopausal women with on average ≥ 14 HF per week  SERT 50/100 mg/d or PLA | | | | | 2/6 weeks | | | | HF frequency, severity score | Similar decrease in HF frequency PLA=38%, SERT=39% (p=0.94); HF score PLA=41% SERT=42% (p=0.86) |
| Kerwin, 2007 | 102 | | RCT | | Double-blind, PLA-controlled, cross-over | | | | | | Menopausal women  SERT 50 mg/d or PLA | | | | | 9 weeks | | | | HF frequency, severity | Significant but clinically modest reduction in frequency and HF index (frequency x severity) among some (1/3) women; no change in 1/3, symptoms worsened in 1/3 |
| Aedo 2011 | 44 | | RCT | | Double-blind, PLA-controlled | | | | | | Post-menopausal women  50 mg/day SERT vs. PLA | | | | | 12 weeks | | | | HF symptoms | Symptom improvement: PLA 35.3%, 81.3% SERT (p=0.01) |
|  |  | |  | |  | | | | | |  | | | | |  | | | |  |  |
| Kimmick, 2006 | 62 | | RCT | | Double-blind PLA-controlled, cross-over | | | | | | **Breast cancer survivors** on adjuvant tamoxifen therapy with at least 1 HF per day;  SERT 50 mg/d or PLA | | | | | 6 weeks,  Follow-up 12 weeks | | | | HF frequency, severity score | 50% decrease in HF frequency in 36% patients in SERT group; 27% in PLA group (p=0.7) |
| Wu, 2009 | 65 | | RCT | | Double-blind PLA-controlled, cross-over | | | | | | **Breast cancer survivors** and patients at high risk with > 15 HF per week,  25 mg/d SERT titrated up to 100 mg/d vs. PLA | | | | | 6 weeks (4 *verum* treatment weeks) | | | | HF frequency, severity score | No demonstrable efficacy of decreasing HF symptoms with sertraline |
| **Fluoxetine (FLU)** | | | | | | | | | | | | | | | | | | | | | |
| Suvanto-Luukkonen, 2005 | 150 | | RCT | | Double-blind, PLA-controlled | | | | | | Post-menopausal women  FLU vs. CT (both at doses of 10 mg: 4 weeks;  20 mg: 8 weeks;  30 mg: 24 weeks)  vs. PLA | | | | | 36 weeks | | | | HF frequency, severity score (Kuperman index) | No significant differences between PLA and both SSRIs (HF frequency and severity score) |
| Oktem, 2007 | 120 | | RCT | | Active-controlled | | | | | | Post-menopausal women  40 mg isopropanolic CRE vs. 20 mg FLU | | | | | 24 weeks | | | | HF symptom score | After 12 weeks, HF score decreased significantly (p=0.02) in the CRE group compared with that in the FLU group. At 24 weeks, CRE significantly reduced HF (85%) better than FLU (62%) and was also a superior treatment for night sweats (both p<0.001). |
|  |  | |  | |  | | | | | |  | | | | |  | | | |  |  |
| Loprinzi, 2002 | 81 | | RCT | | Double-blind, PLA-controlled, cross-over | | | | | | **Breast cancer survivors** with at ≥ 14 HF per week,  FLU 20 mg/day or PLA | | | | | 4 weeks | | | | HF score | Significant reduction in HF score: 50% (FLU), 36% (PLA), p=0.02 |
| **Citalopram (CT)** | | | | | | | | | | | | | | | | | | | | | |
| Suvanto-Luukkonen, 2005  (cf. Fluoxetine above) | 150 | | RCT | | Double-blind, PLA-controlled | | | | | | Post-menopausal women  FLU vs. CT (both at doses of 10 mg: 4 weeks;  20 mg: 8 weeks;  30 mg: 24 weeks)  vs. PLA | | | | | 36 weeks | | | | HF frequency, severity score (Kuperman index) | No significant differences between PLA and both SSRIs (HF frequency and severity score) |
| Barton, 2010 | 254 | | RCT | | Double-blind, PLA-controlled | | | | | | Post-menopausal women with at ≥ 14 HF per week  CT at doses of 10, 20, or 30 mg/d versus PLA | | | | | 6 weeks | | | | HF frequency and score | Significant reduction in frequency (46%, 43%, 50%) and score (49%, 50%, 55%) for the 10, 20, and 30 mg CT dose, vs. PLA (20% and 23%), respectively; p<0.001 and p<0.002 |
|  |  | |  | |  | | | | | |  | | | | |  | | | |  |  |
| Barton, 2003 | 26 | | Uncon-trolled | | Uncontrolled | | | | | | **Breast cancer survivors** with at ≥ 14 HF per week  10 mg CT: week 1  20 mg CT: weeks 2-4 | | | | | 4 weeks | | | | HF frequency and score | Reduction in mean HF frequency (58%) and score (64%) from baseline |
| **Escitalopram (SCT)** | | | | | | | | | | | | | | | | | | | | | |
| Defronzo, 2009 | 25 | | OBS | | Uncontrolled | | | | | | Menopausal women with at ≥ 14 HF per week  10-20 mg/d ECT | | | | | 8 weeks | | | | HF frequency, severity score | Significant decreases in both HF frequency (52.2%) and severity (53.8%) (both p=0.0001) |
| Freedman, 2011 | 42 | | 2 RCT | | Double-blind, PLA-controlled | | | | | | Postmenopausal women with on average 20 to 20.6 HF per day  Study 1:10 mg/d ECT or PLA (N=16)  Study 2: 20 mg/d ECT or PLA (N=26) | | | | | 8 weeks | | | | HF frequency | ECT at 10 mg or 20 mg/day was not effective in treating menopausal HF |
| Freeman 2011  Carpenter, 2012  Ensrud, 2012 | 205 | | RCT | | Double-blind, PLA-controlled, parallel arm | | | | | | Peri- and post-menopausal women with at ≥ 28 HF per week  ECT 10-20 mg/d or PLA  Stratified by race | | | | | 8 weeks + 3 weeks follow-up | | | | HF frequency, severity score | Significantly greater reduction than PLA in HF frequency (p=0.004) and severity (p=0.003) |
|  |  | |  | |  | | | | | |  | | | | |  | | | |  |  |
| **Venlafaxine (VEN)** | | | | | | | | | | | | | | | | | | | | | |
| Evans, 2005 | 80 | | RCT | | | Double-blind, PLA-controlled | | | | Post-menopausal women with at ≥ 14 HF per week  37.5 mg/d VEN ER for 1 week followed by 75 mg/d VEN SR for 11 weeks or PLA | | | | | 12 weeks | | | | | HF score | A trend toward lower HF score was seen in treatment group (p=0.25). Significantly more AEs in VEN group vs. PLA: dry mouth (81% vs. 44%), sleeplessness (88% vs. 47%, decreased appetite (81% vs. 53%) |
| Loprinzi, 2006 | 218 | | RCT | | | Active-controlled | | | | Post-menopausal women with at ≥ 14 HF per week  MPA 400 mg i.m. vs.  37.5 mg / 75 mg/d VEN | | | | | 6 weeks | | | | | HF frequency and score | 50% decrease in HF frequency reported by 46% (VEN) and 74% (MPA) and HF score decreased by 55% (VEN) versus 79% (MPA) (all p<0.0001), with less toxicity in the MPA arm. |
|  |  | |  | | |  | | | |  | | | | |  | | | | |  |  |
| Loprinzi, 1998 | 31 | | OBS | | | Uncontrolled | | | | Post-menopausal women  including **breast cancer survivors** (82%) and **prostate cancer survivors** with androgen-deprivation therapy (18%) with ≥ 2 HF per day,  12.5 mg/d VEN b.i.d. | | | | | 4 weeks | | | | | HF frequency and score | 54% of patients reported ≥50% decrease in number of HF and 58% reported a median 55% reduction in HF score (95% CI 22-71%). Two drop-outs due to AEs (decreased concentration, depression, nausea, dry mouth, fatigue, and sleepiness) |
| Carpenter, 2007 | 77 | | 2 RCT | | | 2 double-blind, PLA-controlled cross-over trials | | | | **Breast cancer survivors** with ≥ 1 HF per day,  Low dose: 37.5 mg/d VEN  (n=57)  High dose: 75 mg/d VEN  (n=20) | | | | | 2 x 6 weeks | | | | | Frequency, severity, impact on life | Compared to PLA, low dose and high dose evoked modest acute reductions in HF frequency (-42% and –25%, (both p=0.001) and severity (-7% and -27% (both p<0.001) with few side effects (sleep disturbance, constipation, dry mouth, headache) |
| Quella, 1999 | 23 | | OBS | | | Uncontrolled | | | | **Prostate cancer survivors** with ≥ 14 HF per week,  VEN 12.5 mg b.i.d. | | | | | 4 weeks | | | | | Frequency and severity of HF | From baseline to week 4, 38% reported at least a 50% decrease in HF frequency and 63% of patients showed a median 54% decrease in weekly HF score. |
| Loprinzi, 2000 | 229 | | RCT | | | Double-blind,  PLA-controlled, randomised | | | | **Breast cancer survivors** with ≥ 14 HF per week,  37.5 mg/d, 75 mg/d or 150 mg/d VEN vs. PLA | | | | | 4 weeks | | | | | HF frequency and score | HF frequencies and scores reduced from baseline by 19 and 27% (PLA), 30 and 37% (37.5 mg), 46 and 61% (75 mg) and 58 and 61% (150 mg) (all p<0.001). Significantly more AEs (mouth dryness, decreased appetite, nausea, constipation) in the 75 mg and 150 mg VEN groups vs. PLA. |
| Loibl, 2007 | 80 | | RCT | | | Double-blind | | | | **Breast cancer survivors** with ≥ 2 HF per day,  37.5 mg/d VEN b.i.d.  0.075 mg CD b.i.d vs. | | | | | 4 weeks | | | | | HF frequency | VEN is significantly (p=0.025) more effective in reducing the frequency of HF. Significantly (p=0.05) more nausea in the VEN groups |
| Buijs, 2009 | 60 | | RCT | | | Double-blind, cross-over | | | | **Breast cancer survivors** with ≥ 14 HF per week,  75 mg/d VEN vs. 0.05 mg CD b.i.d | | | | | 8 weeks | | | | | HF frequency and severity | Median reduction in HF score was comparable, 49% for VEN and 55% for CD (ns). Dropouts due to toxicity: VEN:14/59 CD:5/53 (p=0.038) |
| Boekhout, 2011 | 102 | | RCT | | | Double-blind, PLA-controlled, | | | | **Breast cancer survivors** with ≥ 2 HF per day,  75 mg/d VEN vs.  0.1 mg/d CD vs. PLA | | | | | 12 weeks | | | | | HF score | Overall, during the 12 weeks, HF scores were significantly lower in the CD and VEN group vs. PLA (p<0.001 and p=0.045, respectively); but when compared to PLA at week 12 specifically, only CD showed lower HF scores (p=0.03); VEN (p=0.07). Nausea (p=0.02), constipation (p=0.04) higher with VEN |
| Bordeleau, 2010 | 66 | | RCT | | | Group-sequential, open-label, randomised, cross-over trial | | | | **Breast cancer survivors** with ≥ 14 HF per week,  37.5/75 mg/d VEN vs. 300/600/900 mg/d GP | | | | | 4 weeks | | | | | HF score | Both agents reduced HF score by about 66% (p<0.001). 32% preferred GP, 68% VEN. VEN showed more nausea, appetite loss, constipation, and reduced negative mood changes than GP, GP had more dizziness and better appetite (all p<0.05) |
| Vitolins, 2013 | 120 | | RCT | | | Double-blind, PLA-controlled | | | | **Prostate cancer survivor**s with ≥ 4 HF per day,  PLA + milk protein vs. VEN 75 mg/d + milk protein vs. PLA + 160 mg/d soy ISOF vs. VEN 75 mg/d + 160 mg/d soy ISOF | | | | | 12 weeks | | | | | HF frequency and severity | Neither VEN nor soy ISOF were effective with regard to HF frequency and severity |
| **Desvenlafaxine (DESV)** | | | | | | | | | | | | | | | | | | | | | |
| Speroff, 2008 | 707 | RCT | | | | Double-blind PLA-controlled | | | | Post-menopausal women with ≥ 50 moderate to severe HF per week,  50, 100, 150, and 200 mg/d DESV or PLA | | | | | 12 weeks | | | | | HF frequency and severity | Highest reduction in frequency compared with PLA with 100 mg/d at week 12 (p=0.005). DESV-treated women reported significantly more treatment-related AEs than PLA-treated women (first treatment week only) |
| Archer, 2009a | 567 | RCT | | | | Double-blind, PLA-controlled | | | | Post-menopausal women with ≥ 50 HF per week,  100 mg/d or 150 mg/d DESV vs. PLA | | | | | 26 weeks | | | | | HF frequency and severity | Significantly reduced frequency with 100 and 150 mg DESV vs. PLA (60%, 66% and 47%, p≤0.002) after 12 weeks. After 26 weeks only significant reduction with 150 mg. Significantly more DESV-treated subjects than PLA discontinued because of AEs during week 1 only |
| Archer, 2009b  and Cheng, 2013 | 458 | RCT | | | | Double-blind, randomised, PLA-controlled | | | | Menopausal women with ≥ 50 moderate to severe HF per week,. 100 or 150 mg/d DESV vs. PLA | | | | | 12 weeks | | | | | HF frequency and severity | Reduced frequency vs. PLA of 66.6% vs. 50.8, respectively (p<0.012) and reduced severity (p<0.048) |
| Bouchard, 2012 | 485 | RCT | | | | Double-blind, PLA-controlled | | | | Post-menopausal women with ≥ 50 moderate to severe HF per week,  100 mg/d DESV, 2.5 mg/d tibolone vs. PLA | | | | | 12 weeks | | | | | HF frequency | At week 12, no significant effect of DESV vs. PLA. Significantly (p<0.001) reduced HF frequency in tibolone group vs. PLA |
| Pinkerton, 2013 | 365 | RCT | | | | Double-blind, PLA-controlled | | | | Post-menopausal women with ≥ 50 moderate to severe HF per week,  100 mg/d DESV vs. PLA | | | | | 12 week / one year | | | | | HF frequency and severity | At week 12, DESV reduced significantly vs. PLA frequency and severity score by 62% (PLA 38%, p<0.001) and 25% (PLA 12%, p<0.001). More DESV patients discontinued treatment (p=0.016) |
| **Mirtazapine (MRT)** | | | | | | | | | | | | | | | | | | | | | |
| Perez, 2004 | 22 | | OBS | | | Uncontrolled trial | | | | Menopausal women including 59% **breast cancer survivors** with ≥ 14 HF per week,7.5 (wash-in), 15 or 30 mg/d MRT | | | | | 4 weeks | | | | | HF frequency and severity | Median reduction of HF frequency and score of 52.5% and 59.5%, respectively. |
| Biglia, 2007 | 40 | | OBS | | | Uncontrolled trial | | | | **Breast cancer survivors** with ≥ 7 HF per day,  30 mg/d MRT | | | | | 12 weeks | | | | | HF score | 55.6% reduction of HF frequency and 61.9% reduction in HF score from baseline (both p<0.05). Seven patients discontinued due to somnolence |
| **Bupropion (BU)** | | | | | | | | | | | | | | | | | | | | | |
| Perez, 2006 | 21 | | OBS | | | Uncontrolled | | | | **Breast and prostate cancer survivors**  7 men and 13 women with ≥ 14 HF per week,  BU: 150 mg daily (first 3 days) and then 150 mg b.i.d. | | | | | 4 weeks | | | | | HF frequency | There was no greater reduction in HF frequency and/or severity than that which would be expected with a PLA |
| **Moclobemide (MOC)** | | | | | | | | | | | | | | | | | | | | | |
| Tarim, 2002 | 30 | | RCT | | | Double-blind, PLA-controlled | | | | Post-menopausal women with ≥ 14 HF per week,  moclobemide 150 mg daily vs. moclobemide 300 mg daily vs. PLA | | | | | 5 weeks | | | | | HF score | Reductions in the HF severity score were 24.4% (PLA) group, 69.8% (150 mg), and 35.0% (300 mg). |

| **Vitamin E** | | | | | | | |
| --- | --- | --- | --- | --- | --- | --- | --- |
| Ziaei, 2007 | 51 | RCT | Double-blind PLA-controlled, cross-over | Menopausal women with ≥ 2 HF per day, 400 IU/d vitamin E vs. PLA | 4 weeks active, 1 week washout, 4 weeks PLA | HF frequency, severity, score | HF frequency and HF score decreased significantly with vitamin E vs. PLA (p<0.0001). |
| Barton, 1998 | 120 | RCT | Double-blind PLA-controlled, cross-over | **Breast cancer survivors** with ≥ 14 HF per week, on Vitamin E (800 IU/d) vs. PLA | 4 weeks | HF frequency, severity, score | Although treatment with vitamin E preparations significantly reduced HF frequency vs. PLA, the effect was small (1 HF less per day) and therefore not clinically relevant |
| **Phyto-oestrogens – Isoflavones (ISOF)  Results from this section are cited in the order of positive results, inconclusive results and negative results, irrespective of the comparator.** | | | | | | | |
| Albertazzi, 1998 | 104 | RCT | Double-blind, PLA-controlled | Post-menopausal women with ≥ 7 moderate to severe HF per day,  40 g isolated soy protein vs. 60 g PLA (casein) | 12 weeks | HF frequency | After, 4, 8, and 12 weeks of treatment, soy protein significantly reduced (p<0.01) the number of HF |
| Scambia, 2000 | 39 | RCT | Double-blind, PLA-controlled | Post-menopausal women  50 mg ISOF daily vs. PLA | 6 weeks | HF frequency, severity score | Significant (p<0.001) reduction in HF frequency (about 40%) and severity score |
| Han, 2002 | 80 | RCT | Double-blind, PLA-controlled | Post-menopausal women with presence of HF,  100 mg ISOF/d vs. PLA | 16 weeks | HF frequency, severity score | ISOF evoked a decrease in HF score from baseline (p<0.01), and compared to PLA (p<0.01). |
| van de Weijer, 2002 | 30 | RCT | Double-blind, PLA-controlled | Post-menopausal women with ≥ 5 HF per day,80 mg ISOF/d vs. PLA | 4 weeks PLA followed by 12 weeks treatment | HF frequency, severity score | During the 4 weeks of PLA, HF frequency decreased by 16%. Subsequently, a 44% significant decrease was seen in ISOF group (p<0.01) while no further reduction occurred in the PLA group (p=0.0154) |
| Jeri, 2002 | 30 | RCT | Double-blind, PLA-controlled | Post-menopausal women with ≥ 5 HF per day,40 mg/d standardised ISOF vs. PLA | 16 weeks | HF frequency and severity | Compared to PLA, ISOF reduced frequency (10.5% vs. 48.5%; p<0.001) and severity (0% vs. 47%; p<0.001) |
| Sammartino, 2003 | 70 | RCT | Open, PLA-controlled | Post-menopausal women with ≥ 7 moderate to severe HF per day,36 mg/d genistein vs. 3.3 g/d calcium phosphate and cholecalciferol  8 mg/day (control) | 48 weeks | HF severity index | After 24 or 48 weeks treatment, HF severity index was significantly (p<0.05) lower in the ISOF than in the PLA group. No difference on endometrial thickness in either group |
| Nahas, 2004 | 50 | RCT | Double-blind, PLA controlled | Post-menopausal women  60 mg/d soy germ ISOF vs. PLA | 24 weeks | HF frequency, severity score | After 16 and 24 weeks, ISOF showed a significantly reduced HF score (p<0.05). Reductions were 51.4% and 57.1% in the ISOF group and 18.8% and 18.8% in the PLA group. |
| Nahas, 2007 | 80 | RCT | Double-blind, PLA-controlled | Post-menopausal women with ≥ 5 HF per day,  100 mg/ soy ISOF vs. PLA | 40 weeks | HF frequency, severity | After 40 weeks treatment HF frequency decreased significantly more (p<0.001) in the ISOF group. Severity decreased by 69.9% (ISOF) and 33.7 (PLA) (p<0.001) |
| Khaodhiar, 2008 | 190 | RCT | Double-blind, PLA-controlled | Post-menopausal women with ≥ 4 ≤ 15 HF per day,  40 or 60 mg/d DRI (ISOF) vs. PLA | 12 weeks | HF frequency, severity | 40 and 60 mg of DRI equally and significantly improved HF frequency and severity at 12 weeks compared to PLA (52%, 51% vs. 39%, respectively). |
| Cheng, 2007 | 60 | RCT | Double-blind, PLA-controlled | Post-menopausal women  60 mg ISOF daily vs. PLA | 12 weeks | HF score | At week 12, HF score was significantly (p<0.01) lower in the ISOF group (57%) compared to baseline and to those in the PLA group (p<0.01). No difference was found in the PLA group compared to baseline. |
| Radhakrishnan, 2009 | 100 | RCT | Double-blind, PLA-controlled | Post-menopausal women  75 mg/d soy ISOF vs. PLA | 24 weeks | HF score | Significant improvement of HF score in both groups (p<0.05), but ISOF group showed a significantly greater improvement (p<0.05) |
| Ye, 2012 | 90 | RCT | PLA-controlled | Post-menopausal women  84 mg/d vs. 126 mg/d soy germ ISOF vs. PLA | 24 weeks | HF frequency and severity | HF frequency and severity decreased in all treatment groups. However the percentage decrease was greater (p<0.01) in the two ISOF groups. |
| Aso, 2012 | 160 | RCT | Double-blind, PLA-controlled | Post-menopausal women with ≥ 1 HF per day  10 mg S-(-) equol (an active metabolite of the isoflavone, daidzein) daily vs. PLA | 12 weeks | HF frequency | A greater decrease from baseline in HF frequency was seen in the S-(-) equol group compared to PLA (- 58.7%, vs.-34.5%, p=0.009). |
| Mainini, 2013 | 150 | RCT | Randomised, PLA-controlled | Post-menopausal women with ≥ 20 HF per week,  60.8 mg red clover ISOF plus 19.2 mg soy ISOF vs. PLA | 12 weeks | HF frequency and score | HF frequency and score were significantly lower than PLA (both p<0.05) after 3 months |
| D’Anna, 2007/2009 | 389 | RCT | Double-blind, PLA-controlled | Post-menopausal women  54 mg/genistein vs. PLA | 12 months extended to 24 months | HF frequency, severity score | Compared to PLA, significant reduction in HF frequency and severity after 12 months by 56.4% and 37.5%, respectively (each p<0.001). No further decrease in HF frequency after 24 months |
| Ferrari, 2009 | 180 | RCT | Double-blind, PLA-controlled | Menopausal women with ≥ 5 moderate to severe HF per day,  80 mg/d ISOF (60 mg/d genistein) vs. PLA | 12 weeks | HF frequency and severity | Significantly greater reduction in HF frequency after 12 weeks (p=0.023). However, HF severity index decreased in both groups with no significant difference between groups |
| Evans, 2011 | 84 | RCT | Double-blind, PLA-controlled | Post-menopausal women with ≥ 40 HF per week,  30 mg/d genistein vs. PLA | 12 weeks | HF frequency, severity and duration | Compared to PLA, significantly (p=0.01) fewer HF and decreased HF duration/day (p=0.009) after genistein treatment at week 12, however, no significant differences in severity were observed between treatments |
| Murkies, 1995 | 58 | RCT | Double-blind, controlled | Post-menopausal women with ≥ 14 HF per week,  45 g flour daily of dietary supplements (soy vs. wheat) | 12 weeks | HF score | HF scores decreased significantly (p<0.001) from baseline with soy (41.7%) and wheat (24.5%), but no significant difference between treatments |
| Crisafulli, 2004 | 90 | RCT | Double-blind, active-controlled | Post-menopausal women  estrogen-progestogen  therapy (EPT) 1 mg/d 17β-oestradiol + 0.5 mg/d norethi­sterone acetate vs. 54 mg/d genistein vs. PLA | 52 weeks | HF frequency, severity score | Compared to PLA, genistein reduced daily HF scores significantly after 52 weeks by a mean of 24% (95% CI: −43 to −5%; p<0.01) and EPT by 54% (95% CI: −74 to −33%; p<0.001), as compared with PLA. |
| Labos, 2013 | 89 | RCT | Randomised, actively controlled | Post-menopausal women  644 mg/d DT56a from soybeans or 17β-estradiol 1 mg plus norethisterone acetate 0.5 mg vs. no treatment | 52 weeks | HF severity score | Both HT and DT56a significantly decreased HF score compared to no treatment (p<0.001 and p=0.013). |
| Baber, 1999 | 51 | RCT | Double-blind PLA-controlled, cross-over | Post-menopausal women with ≥ 3 HF per day,  40 mg ISOF/d vs. PLA | 12 weeks | HF frequency, severity score | After 12 weeks, no significant difference was observed between active and PLA groups in reducing HF |
| Knight, 1999 | 37 | RCT | Double-blind, PLA-controlled | Post-menopausal women with ≥ 3 HF per day,  40 mg or 160 mg ISOF/d vs. PLA | 12 weeks | HF frequency, severity score | No significant difference in HF frequency and severity score among the groups. |
| Upmalis, 2000 | 177 | RCT | Double-blind, PLA-controlled | Post-menopausal women with ≥ 5 HF per day  Soy ISOF extract (containing 50 mg genistin and daidzin/d) vs. PLA | 12 week | HF frequency, severity score | Decreases in HF frequency and severity were observed within 2 weeks in the soy group; no relief was seen in the PLA group for the first 4 weeks. Significant differences were noted after 6 weeks (p=0.03) but not after 12 weeks (p=0.08) |
| St Germain, 2001 | 69 | RCT | Double-blind, controlled | Peri-menopausal women with ≥ 10 HF or night sweats per week,  ISOF-rich soy protein vs. ISOF-poor soy protein vs. whey control protein | 24 weeks | HF frequency, severity score | Neither isoflavone-rich nor isoflavone-poor soy protein provided relief of vasomotor symptoms |
| Burke, 2003 | 241 | RCT | Double-blind, active-controlled | Peri- and post-menopausal women with ≥ 1 HF per day,  25 g of soy protein alone (control) vs. plus either 42 mg ISOF/d or plus 58 mg ISOF/d | 24 months | HF frequency, severity score | No significant differences in the number and severity of HF  among the high ISOF, middle ISOF, or control groups. |
| Faure, 2002 | 75 | RCT | Double-blind, PLA-controlled | Post-menopausal women with ≥ 7 moderate to severe HF (including night sweats) per day,  70 mg ISOF/d (genistin, daidzein) vs. PLA | 16 weeks | HF frequency | At week 16, daily HF reduction of 61% was observed in the ISOF group, and 21% with PLA, ITT analysis of treatment effects between groups: p=0.01. Many withdrawals in study mitigate positive results. |
| Campagnoli, 2005 (Study A) | 29 | RCT | Double-blind, PLA-controlled, cross-over | Post-menopausal women with ≥ 5 HF moderate to severe HF per day,  60 mg ISOF daily vs. PLA | 12 weeks | HF frequency, severity score | Compared to PLA, ISOF extract did not show greater efficacy |
| Tice, 2003 | 252 | RCT | Double-blind, PLA-controlled | Post-menopausal women with ≥ 35 HF per week,  82 mg/d ISOF vs. 57 mg ISOF vs. PLA | 12 weeks | HF frequency | Reductions in HF frequency at week 12 were similar in all groups. Neither supplement exerted significant effects on HF. |
| Penotti, 2003 | 62 | RCT | Double-blind, PLA-controlled | Post-menopausal women with ≥ 7 HF per day,  72 mg ISOF vs. PLA | 24 weeks | HF frequency, severity score | Both treatments reduced number of HF by 40%. ISOF was no more effective than PLA in reducing HF. |
| Secreto, 2004 | 388 | RCT | Double-blind, double-dummy, PLA controlled | Post-menopausal women  40 mg ISOF twice daily + 3 mg melatonin (evening) vs. 40 mg ISOF twice daily vs. 3 mg melatonin (evening) vs. PLA | 12 weeks | HF score | No advantage of ISOF or melatonin over PLA for the relief of HF symptoms |
| Lewis, 2006 | 99 | RCT | Double-blind, controlled | Post-menopausal women, 25 g flaxseed (50 mg lignans) daily vs. 25 g soy (42 mg ISOF) daily vs. wheat (PLA) | 16 weeks | HF frequency, severity score | There was no significant effect on HF symptoms by either dietary flaxseed or soy flour |
| D’Anna, 2007/2009 | 389 | RCT | Double-blind, PLA-controlled | Post-menopausal women  54 mg/genistein vs. PLA | 12 months extended to 24 months | HF frequency, severity score | Compared to PLA, significant reduction in HF frequency and severity after 12 months by 56.4% and 37.5%, respectively (each p<0.001). No further decrease in HF frequency after 24 months |
| Ferrari, 2009 | 180 | RCT | Double-blind, PLA-controlled | Menopausal women with ≥ 5 moderate to- severe HF per day 80 mg/d ISOF (60 mg/d genistein) vs. PLA | 12 weeks | HF frequency and severity | Significantly greater reduction in HF frequency after 12 weeks (p=0.038). However, HF severity index decreased in both groups with no significant difference among groups |
| Evans, 2011 | 84 | RCT | Double-blind, PLA-controlled | Post-menopausal women with ≥40 HF per week  30 mg/d genistein vs. PLA | 12 weeks | HF frequency, severity and duration | Compared to PLA, significantly (p=0.01) fewer HF and decreased HF duration/day (p=0.009) after genistein treatment at week 12, however, no significant differences in severity were observed between treatments |
| Levis, 2011 | 248 | RCT | Double-blind, PLA-controlled | Menopausal women  200 mg soy ISOF daily vs. PLA | 2 years | HF frequency | Significantly more patients (p=0.02) (48.4%) in the soy group experienced HF than in the PLA group (31.7%). |
|  |  |  |  |  |  |  |  |
|  |  |  |  |  |  |  |  |
| Quella, 2000 | 177 | RCT | Double-blind PLA-controlled, cross-over | **Breast cancer survivors** with ≥ 14 HF per week,  150 mg soy/d ISOF vs. PLA | 4 weeks per arm | HF frequency | There was no beneficial effect on HF frequency observed after soy phyto-oestrogen treatment |
| Van Patten, 2002 | 123 | RCT | Double-blind, PLA-controlled | **Breast cancer survivors** with ≥ 10 HF per week  Soy beverage (90 mg ISOF/d) vs. PLA | 12 weeks | HF frequency, severity score | No significant effect on HF frequency and severity |
| Nikander, 2003 | 62 | RCT | Double-blind, PLA-controlled, cross-over | **Breast cancer survivors,** 114 mg ISOF vs. PLA | 12 weeks | HF score | HF score were reduced by 15.5% (ISOF) compared to 14.7% (PLA). There were no significant differences between treatments. |
| MacGregor, 2005 | 72 | RCT | Double-blind, PLA-controlled | **Breast cancer survivors**  70 mg ISOF daily vs. PLA | 12 weeks |  | There was no statistical difference in menopausal symptom scores |
| Sharma, 2009 | 33 | RCT | Double-blind, PLA-controlled | **Prostate cancer survivors**  160 mg ISOF from soy protein vs. PLA from whole milk protein | 12 weeks | HF score | At 12 weeks, there were no significant differences between the 2 groups in any outcome measure |
|  | | | | | | | |

| ***Melatonin*** | | | | | | | | | | | | |
| --- | --- | --- | --- | --- | --- | --- | --- | --- | --- | --- | --- | --- |
| Chen, 2014 | | 95 | | RCT | | Double-blind, PLA-controlled | | **Breast cancer survivor,** with on average 2.3 HF per day.  3 mg/d melatonin vs. PLA | | 16 weeks | HF frequency and severity | Improvement in subjective sleep quality but no significant decrease in HF frequency or severity between treatments |
|  | |  | |  | |  | |  | |  |  |  |
| ***Hops*** | | | | | | | | | | | | |
| Heyerick, 2006 | | 67 | | RCT | | Double-blind, PLA-controlled | | Menopausal women with 2–5 HF per day  Hop extract standardised to 100 or 250 μg 8-PN daily vs. PLA | | 12 weeks | HF frequency, severity score | All groups showed a significant reduction in HF score after 6 and 12 weeks. The 100 μg 8-PN hop extract was significantly superior to PLA after 6 weeks (p=0.023) but not after 12 weeks (p=0.086). At both time points, the 250 μg high dose was less active than the lower dose. |
| Erkkola, 2010 | | 36 | | RCT | | Double-blind, PLA-controlled, cross-over | | Post-menopausal women  100 μg 8-PN daily | | 8 weeks | HF score | Overall, there were no significant differences between the treatments in HF score (p=0.10) |
|  | |  | |  | |  | |  | |  |  |  |
| **Red clover (RC)** | | | | | | | | | | | | |
| Hidalgo, 2005 | | 60 | | RCT | | Double-blind, PLA-controlled, cross-over | | Post-menopausal women  80 mg/d red clover ISOF vs. PLA | | 12 weeks | HF frequency, severity score | HF score decreased significantly after each treatment, and more pronounced after ISOF  (baseline: 27.2±7.7; after ISOF: 5.9±3.9; after PLA: 20.9±5.3, p<0.05). |
| Geller, 2009 | | 89 | | RCT | | Double-blind, PLA-controlled | | Peri- and post-menopausal women with ≥ 35 HF per week  ,  128 mg/d ethanolic CRE vs. 378 mg/d ethanolic red clover extract vs. 0.625 mg CEE + 2.5 mg MPA daily vs. PLA | | 52 weeks | HF frequency | Reductions in HF frequency and severity by CRE (34%), red clover (57%) did not differ significantly from PLA (63%), though CEE/MPA (94%) did. |
| Lipovac, 2012 | | 109 | | RCT | | PLA-controlled, cross-over | | Post-menopausal women with > 5 HF per day  80 mg/d red clover ISAF vs. PLA | | 12 weeks | HF frequency and severity | When ISOF was given first, HF frequency and severity decreased by about 70% compared to baseline (p=0.001). When given after PLA, ISOF effects were slightly smaller but still significant (p=0.001). |
| **Flaxseed *(Linum usitatissimum)*** | | | | | | | | | | | | |
| Lewis, 2006 | | 99 | | RCT | | Double-blind, controlled | | Post-menopausal women  25 g flaxseed (50 mg lignans) daily vs. 25 g soy (42 mg ISOF) daily vs. wheat (PLA) | | 16 weeks | HF frequency, severity score | There was no significant effect on HF symptoms by either dietary flaxseed or soy flour |
| Colli, 2012 | | 90 | | RCT | | PLA-controlled | | Menopausal women  1000 mg/d flaxseed extract (100 mg SDG) vs. 90 mg/d ground whole flaxseed vs. PLA | | 24 weeks | HF index, intensity | HF index decreased compared to baseline after flaxseed extract (p=0.007) and flaxseed meal (P=0.005), but not after PLA. At the end of treatment, no significant differences were observed between treatments. |
|  | |  | |  | |  | |  | |  |  |  |
| Pruthi, 2012 | | 188 | | RCT | | PLA-controlled | | Post-menopausal women and **breast cancer survivors** (ca. 50%) with ≥ 28 HF per week  flaxseed (providing 410 mg lignans) vs. PLA | | 6 weeks | HF frequency, severity score | There was no statistical difference in HF frequency or score between the treatments (p=0.29). |
| **St. John’s wort (*Hypericum perforatum***) (HP) | | | | | | | | | | | | |
| Al-Akoum, 2009 | | 47 | | RCT | | Double-blind, PLA-controlled | | Peri-menopausal women with ≥ 3 HF per day,  900 mg ethanolic HP extract t.i.d. vs. PLA | | 12 weeks | HF frequency, severity score | A non-significant difference  in favour of the HP group was observed in the daily HF frequency and the HF score. |
| Abdali, 2010 | | 100 | | RCT | | Double-blind, PLA-controlled | | Menopausal women with ≥ 1 moderate to severe HF per day;  20 HP drops t.i.d. corresponding to 0.2 mg/ml vs. PLA | | 8 weeks | HF frequency, severity score | HF frequency was reduced by 53.6% (HP) and 31.7% (PLA) (p<0.001). Severity score was reduced by 59.7% (HP) and 26.1% (PLA) (p<0.001). |
| Uebelhack, 2006 | | 301 | | RCT | | Double-blind, PLA-controlled | | Menopausal women  127.5 mg isopropa­nolic CRE ^*)^ plus 1190 mg of an ethanolic HP extract) during week 1-8; 63.75 mg and 595 mg respectively during week 9-16 vs. PLA | | 16 weeks | Symptom score | Symptom score decreased by 50% (HP & CRE extract) and 19.6% (PLA) in the PLA group (p<0.001). |
| Briese, 2007 | | 6141 | | OBS | Prospective, controlled, open-label, observational | | | Menopausal women  Combination therapy (60 mg/d of an isopropanolic CRE ^*)^ plus 595 mg/d ethanolic HP extract ^*)^ vs. monotherapy (40 mg isopropanolic CRE extract) | | 52 weeks | Symptom score | HF score decreased to a similar extent after both treatments by 51.9% (monotherapy, n=3027) and 55.3% (combination therapy, n=3114) |
|  | |  | |  | |  | |  | |  |  |  |
| **Evening primrose *(Oenothera biennis)*** | | | | | | | | | | | | |
| Farzaneh, 2013 | | 56 | | RCT | | PLA-controlled | | Menopausal women with ≥ 4 HF per day  500 mg evening primrose oil vs. PLA | | 6 weeks | HF frequency, severity score | Improvement in HF frequency and severity were 39 and 42%, (evening primrose) compared to 32% and 32% (PLA), respectively. Only severity improvement was significant for evening primrose compared to PLA (p<0.05) |
| **French maritime pine bark (Pycnogenol)** | | | | | | | | | | | | |
| Yang, 2007 | | 200 | | RCT | | Double-blind, PLA-controlled | | Peri-menopausal women  200 mg French maritime pine bark extract vs. PLA | | 24 weeks | HF frequency, severity score | In contrast to PLA, HF severity improved significantly (p<0001). |
| Kohama, 2013 | | 170 | | RCT | | Double-blind, PLA-controlled | | Peri-menopausal women  30 mg pycnogenol from French maritime pine bark extract daily vs. PLA | | 12 weeks | HF frequency, severity score | Compared to baseline, pycnogenol significantly (p < 0.05) improved most HF symptoms, especially vasomotor and insomnia/sleep problems. Total HF score decreased significantly by 56% as compared to PLA (-39%) after 12 weeks of treatment (p < 0.05). |
| **Sibiric Rhubarb *(Rheum rhaponticum)* (RR)** | | | | | | | | | | | | |
| Heger, 2006  Kaszkin-Bettag, 2007 | | 109 | | RCT | | Double-blind, PLA-controlled | | Peri-menopausal women  4 mg RR extract daily vs. PLA | | 12 weeks | HF frequency, severity score | RR extract significantly reduced the frequency as well as severity of HF by 60.3% (RR) and 14.4% (PLA) (p<0.0001) |
| Kaszkin-Bettag, 2009 | | 109 | | RCT | | Double-blind, PLA-controlled | | Peri-menopausal women  4 mg RR extract daily vs. PLA | | 12 weeks | HF frequency, severity score | RR extract caused a significant  reduction in HF number and score from baseline. |
| Hasper, 2009 | | 82 | | OBS | | Observational,  continuation study of | | Peri-menopausal women  4 mg RR extract daily vs. PLA | | 48 weeks | HF frequency, severity score | Treatment effect from previous study (Heger, 2006 ) was maintained over 48 weeks |
| **Valerian root *(Valeriana officinalis)*** | | | | | | | | | | | | |
| Mirabi, 2013 | | 68 | | RCT | | Double-blind, PLA-controlled | | Postmenopausal women  3 x 255 mg valerian roots vs. PLA | | 8 weeks | HF frequency and severity | Valerian decreased both HF frequency and severity from baseline (p<0.001) while PLA did not. The difference between treatments was significant (p<0.001). |
| **Guaraná *(Paullinia cupana)*** | | | | | | | | | | | | |
| Oliveira, 2013 | | 18 | | OBS | | Uncontrolled | | **Breast cancer survivors** 83% (n=15) with menopausal status with ≥14 HF per week,  2 x 50 mg/d of a *Paullinia cupana* extract | | 6 weeks | HF frequency and severity | Compared to baseline treatment decreased HF frequency (p=0.0009) and severity (p<0.0001) |
| ***Magnesium*** | | | | | | | | | | | | |
| Park 2011 | | 29 | | OBS | | Uncontrolled | | **Breast cancer survivors**  83% (n=24) with post-menopausal status with ≥14 HF per week,  400 – 800 mg magnesium oxide | | 4 weeks | HF score | HF frequency and score were reduced compared to baseline by 41.4% (p=0.02) and 50.4% (p=0.04), respectively |
| **Black cohosh *(Cimicifuga racemosa)*** (CR) | | | | | | | | | | | | |
| Drewe, 2013 | | 442 | | OBS | Prospective observational | | | Menopausal women  42.25 -169 mg/d of an ethanolic CRE ^*)^ | | 36 weeks | HF severity score | After 12 and 36 weeks of treatment significant (p < 0.0001) reduction in HF severity score |
| Lopatka, 2007 | | 584 | | OBS | Observational | | | Menopausal women  42.25 mg/d of an ethanolic CRE ^*)^ | | 16 weeks | HF score | HF score decreased significantly (p<0.0001) by 60% from baseline after 16 weeks of treatment. |
| Vermes, 2005 | | 2016 | | OBS | Observational | | | Menopausal women  40 mg/d of an isopropa­nolic CRE ^*)^ | | 12 weeks | HF score | HF score decreased significantly compared to baseline (p<0.001) |
| Liske, 2002 | | 152 | | RCT | Double-blind, parallel groups | | | Peri- and post-menopausal women received an isopropanolic CRE ^*)^: 39 mg/d vs. 127.3 mg/d | | 24 weeks | HF score | Reduction in HF score by 70% (39 mg) and 72% (127.3 mg). The higher dose did not exert a significantly greater effect than the lower dose. |
| Frei-Kleiner, 2005 | | 122 | | RCT | Randomised, double-blind, PLA-controlled | | | Peri- or post-menopausal women with ≥3 HF per day,  42 mg ethanolic CRE ^*)^ vs. PLA | | 12 weeks | HF score | Only superiority over PLA in sub-group of patients with at least moderate baseline symptom severity (decrease of 47% (CRE) and 21% (PLA); p<0.018) |
| Schellenberg, 2012 | | 180 | | RCT | Double-blind, PLA-controlled | | | Menopausal women  42.25 or 84.5 mg/d of an ethanolic CRE ^*)^ vs. PLA | | 12 weeks | HF severity score | Compared to PLA significant reduction in HF severity score in a dose-dependent manner from baseline to endpoint (p<0.0001 for 13.0 mg and p=0.0003 for 6.5 mg). |
| Osmers, 2005 | | 304 | | RCT | Double-blind, PLA-controlled | | | Post-menopausal women  40 mg/d isopropa­nolic CRE ^*)^ vs. PLA | | 12 weeks | HF frequency, severity score | The CRE was more effective than PLA in the HF severity (p=0.007) |
| Ross, 2012 | | 304 | | RCT | Double-blind, PLA-controlled | | | Post-menopausal women  40 mg/d of an isopropa­nolic CRE ^*)^ vs. PLA | | 12 weeks | Menopausal symptoms | Menopausal symptoms (MRS) decreased significantly (p<0.001) including HF (p=0.007) |
| Newton, 2006 | | 351 | | RCT | Double-blind, PLA-controlled | | | Peri- or post-menopausal women with ≥ 2 HFper day,  received 160 mg/d of an ethanolic CRE *) vs. multibotanical with black cohosh vs. multibotanical plus soy diet vs. conjugated equine oestrogen 0.625 mg daily with or without 2.5 mg MPA vs. PLA (week 1 to 8 once daily, week 9 to 16 twice daily) vs. PLA | | 1 year | HF frequency, severity score | Herbal regimens did not reduce post-menopausal vasomotor symptoms |
| Geller, 2009 | | 89 | | RCT | Double-blind, PLA-controlled | | | Peri-and post-menopausal women with ≥ 35 HF per week ,  128 mg of an ethanolic CRE ^*)^ vs. 120 mg/d of an ethanolic red clover extract vs. 0.625 mg/d CEE + 2.5 mg/d MPA vs. PLA | | 52 weeks | HF frequency | CRE and red clover did not significantly reduce HF frequency or severity as compared with PLA |
| Stoll, 1987 | | 80 | | RCT | Double-blind, PLA-controlled | | | Menopausal women (46-58 years of age) with ≥ 3 HF per day,  0.625 conjugated oestrogen, 8 mg isopropa­nolic CRE, or PLA | | 12 weeks | HF frequency, severity score; among others | After 12 weeks, significant improvement in all measured parameters with CRE; oestrogen dose was deemed too low, and results were not better than PLA. |
| Wuttke, 2003 | | 62 | | RCT | Double-blind, active-controlled | | | **Post-menopausal women** with ≥ 3 HF per day  40 mg isopropanolic CRE ^*)^ vs. 0.6 mg conjugated oestrogens (CE) vs. PLA | | 12 weeks | HF frequency, severity score | CRE and CE were equipotent and borderline superior to PLA in reducing HF severity (both p=0.051) |
| Nappi, 2005 | | 64 | | RCT | Open, parallel groups | | | Menopausal women with ≥ 5 HF per day,  40 mg isopropanolic CRE ^*)^ vs. low-dose TDE 25 µg oestradiol every 7 days+ 10mg dihydrogesterone/d last 12 days of trial | | 12 weeks | HF frequency, severity score | Both treatments reduced significantly (p<0.001) HF frequency and severity to a comparable extent |
| Bai, 2007 | | 244 | | RCT | Double-blind, active-controlled | | | **Menopausal women**  40 mg of an isopropanolic CRE ^*)^ vs. tibolone 2.5 mg daily | | 12 weeks | HF score | HF score decreased significantly by 69% from baseline score for both treatments. The benefit-risk balance for CRE was significantly (p=0.01)  superior to tibolone. |
| Uebelhack, 2006 | | 301 | | RCT | Double-blind, PLA-controlled | | | **Menopausal women** with climacteric complaints for ≥ 3 months  127.5 mg/d of an isopropa­nolic CRE ^*)^ plus 1190 mg/d of an ethanolic HP extract) during week 1-8; 63.75 mg/d and 595 mg/d respectively during week 9-16 vs. PLA | | 16 weeks | Symptom score | Symptom score decreased by 50% (CRE & HP extract) and 19.6% (PLA) in the PLA group (p<0.001). |
| Briese, 2007 | | 6141 | | OBS | Prospective, controlled, open-label, observational | | | **Menopausal women** Combination therapy (60 mg/d of an isopropanolic CRE ^*)^ plus 595 mg/d ethanolic HP extract ^*)^ vs. monotherapy (40 mg isopropanolic CRE extract) | | 52 weeks | Symptom score | HF score decreased to a similar extent after both treatments by 51.9% (monotherapy, n=3027) and 55.3% (combination therapy, n=3114) |
| Oktem, 2007 | | 120 | | RCT | Active-controlled | | | **Post-menopausal women**  40 mg/d isopropa­nolic CRE ^*)^ vs. 20 mg/d FLU | | 26 weeks | HF symptom score | After 12 weeks, HF score decreased significantly (p=0.02) in the CRE group compared to that in the FLU group. At 24 weeks, reductions were 85% (CRE) and 62% (FLU) (p<0.001) |
| Huang, 2013 | 120 | | RCT | | Active controlled | | **Peri-menopausal depression** 20 mg/d PAR vs. 40 mg/d isopropanolic CRE ^*)^ | | 8 weeks | | HF frequency and severity depression (HAMD) | In both studies, PAR decreased HF frequency and severity at the end of treatment: 9.89 ± 3.76 but not as well as the combined treatment 15.75 ± 5.84 (P<0.01). Likewise, the combined treatment was superior for the HAMD 88.3% vs, 78.3% for combined vs. PAR alone (p<0.05) |
|  | |  | |  |  | | |  | |  |  |  |
| Pockaj, 2004 | | 23 | | OBS | Observational | | | **Post-menopausal women (13 breast cancer survivors)** with ≥14 HF per week,  40 mg/d of an isopropanolic CRE ^*)^ | | 4 weeks | HF score | The reduction in mean daily HF frequency was 50%, while weekly HF scores were reduced by 56% at completion of the study. |
| Jacobson, 2001 | | 85 | | RCT | Double-blind, active-controlled | | | **Breast cancer survivors,** who reported HF daily  Unknown dose and type of CRE ^*)^ vs. PLA | | 8 weeks | HF frequency, severity score | No significant difference between CRE and PLA for number and intensity of HF |
| Pockaj, 2006b | | 132 | | RCT | Double-blind, PLA-controlled, cross-over | | | **Breast cancer survivors** with ≥ 14 HF per week,  40 mg/d of an isopropa­nolic CRE ^*)^ vs. PLA | | 4 weeks | HF frequency, severity score | No significant improvement of HF frequency or severity compared to PLA |
| Hernández Muñoz, 2003 | | 150 | | RCT | Open-label, controlled | | | **Breast cancer survivors,** pre-menopausal status with regular menstruation  and normal duration of cycle, and breast  cancer diagnosis with ER-positive tumor.  Tamoxifen 20 mg/d vs. tamoxifen 20 mg/d + 20 mg/d of ethanolic CRE ^*)^ | | 52 weeks | HF frequency, severity | Compared to tamoxifen alone, the number and severity of HF were reduced in the tamoxifen+CR group. Almost half of the patients of the CRE group were free of HF, while severe HF were reported by 24.4% of CRE patients and 73.9% of the tamoxifen alone group (p<0.01). |
| Rostock, 2011 | | 50 | | OBS | Prospective,  observational | | | **Breast cancer survivors,** 74% (n=35) with post-menopausal status,  treated with tamoxifen 10-40 mg/d and 20–80 mg/d of an isopropanolic CRE ^*)^ | | 26 weeks | HF frequency, severity score | Compared to baseline, HF score decreased significantly (p<0.001) |

AD = anti-depressant; AE = adverse events; CD = clonidine; CIT = citalopram; CEE = conjugated equine oestrogens; SR = Sustained release; CR = *Cimicifuga racemosa*; CRE = *Cimicifuga racemosa* extract; DESV = desvenlafaxine; DRI = daizein-rich isoflavones; ECT = escitalopram; ER = extended release; ISOF = isoflavone; FLU = fluoxetine; GP = gabapentin; HAMD = Hamilton depression scale; HF = hot flushes; HP = *Hypericum perforatum* (St. John’s wort) extract; MGA = megestrol acetate; MPA = medroxyprogesterone acetate; MRT = mirtazapine; OBS = observational study; PAR = paroxetine; PG = pregabalin; 8-PN = 8-prenylnargenin; PLA = placebo; RCT = randomised, controlled trial; RR: *Rheum rhaponticum*; SDG = secoisolariciresinol diglucoside; SERT = sertraline; TDE: Transdermal oestradiol; VEN = venlafaxine;

1. Number of patients randomised; ^*)^ = calculated as herbal drug equivalent
